# Modern synergetic neural network for imbalanced small data classification

**DOI:** 10.1038/s41598-023-42689-8

**Published:** 2023-09-21

**Authors:** Zihao Wang, Haifeng Li, Lin Ma

**Affiliations:** Faculty of Computing, Harbin Institute of Technology, No.92, Xidazhi Street, Nangang District, Harbin, 150001 Heilongjiang China

**Keywords:** Mathematics and computing, Computer science

## Abstract

Deep learning’s performance on the imbalanced small data is substantially degraded by overfitting. Recurrent neural networks retain better performance in such tasks by constructing dynamical systems for robustness. Synergetic neural network (SNN), a synergetic-based recurrent neural network, has superiorities in eliminating recall errors and pseudo memories, but is subject to frequent association errors. Since the cause remains unclear, most subsequent studies use genetic algorithms to adjust parameters for better accuracy, which occupies the parameter optimization space and hinders task-oriented tuning. To solve the problem and promote SNN’s application capability, we propose the modern synergetic neural network (MSNN) model. MSNN solves the association error by correcting the state initialization method in the working process, liberating the parameter optimization space. In addition, MSNN optimizes the attention parameter of the network with the error backpropagation algorithm and the gradient bypass technique to allow the network to be trained jointly with other network layers. The self-learning of the attention parameter empowers the adaptation to the imbalanced sample size, further improving the classification performance. In 75 classification tasks of small UC Irvine Machine Learning Datasets, the average rank of the MSNN achieves the best result compared to 187 neural and non-neural network machine learning methods.

## Introduction

Accurate classification of massive samples, as a landmark accomplishment of deep learning, has achieved excellent performance that compares to or surpasses that of humans. However, its performance is based on the premise of a huge number of data with a balanced distribution, which makes it highly susceptible to the overfitting problem with small^1–4^ and imbalanced^5–7^ data. Some approaches try to mitigate the overfitting problem by data augmentation^8–10^. However, the supplemental data obtained by such methods are only the result of distribution prediction of the existing data, whose accuracy is also highly dependent on the amount of data. Therefore, data-scarce fields still rely on traditional neural networks or non-neural machine learning methods.

Among these traditional methods, the recurrent neural networks (RNN)^11–14^ have promising performances, and the synergetic neural network (SNN)^11^ therein natively supports data distribution adaptation, making it more suitable for classification tasks with small amounts of imbalanced data. RNN has the advantages of a small number of parameters, fast training speed, controllable working process, low data dependence, and high robustness^15,16^, which supports its wide application in small data classification tasks^17^. Although SNN is a relatively outdated RNN model, it still has some theoretical advantages over the latest models. All attractors of the SNN correspond to valid memories, saddle points and the local extreme are limited and can be easily escaped, ensuring the network’s unrivaled robustness^18–20^. In addition, its unique synergetic-based dynamics lead to zero error in convergence results and native imbalanced data adaptation^16,21,22^. The overfitting problem can be effectively reduced by repeatedly applying varied attention parameters to different classes of data during the recurrences of the network.

However, these advantages are masked by the network's frequent association errors, and the optimization space of the parameters is employed to overcome this problem, preventing the network's adaption capability from being fully revealed. Researchers apply SNN in tasks including image retrieval^23^, face recognition^24^, and semantic role labeling^25,26^, yet find that the increased task difficulty significantly elevates the frequency of network association errors. Hu first addresses the problem by adding more parameters for adjustment^27^. Other studies optimize parameters with immune clonal strategy^28^, fuzzy integral^29^, and improved particle swarm optimization^30^. Besides, applying the immune clonal strategy to enhance the orthogonality of memories also helps improve accuracy^31^. However, the ideology of these approaches is to use various learning methods to solve SNN’s working problem. From the dynamical system perspective, the association error stems from the initial state being placed in the wrong basin of attraction in its working procedure. Since the cause of the problem is not revealed, parameter tuning becomes the dominant route. Due to the lack of explicit objectives, optimization methods are often based on genetic algorithms. Such a research route substantially complicates SNN’s application process and occupies the optimization space of the parameters, so task-oriented parameter training is difficult to be introduced simultaneously. These problems lead related research to a standstill.

In this paper, we propose a modern synergetic neural network (MSNN) model to properly apply the advantages of SNN to practical problems. We first address the association error and release the parameter tuning space by defining and remodeling the state initialization method of SNN. Although SNN’s first study and some subsequent studies suggest that its initial state characterizes the similarity between samples and memories^11,23,32–34^, we prove that the initial state does not conform to the principles of a similarity metric. Therefore, we distill the network's method of calculating the initial state and remodel it as a definitive solution. Since the new solution isolates the parameter tuning process, the whole optimization space can be reserved for the task properties. We design an Error BackPropagation (EBP) based attention parameter training method that allows MSNN to be co-trained with other network layers for automatic data distribution adaptation. Experimental results on 75 imbalanced small UC Irvine Machine Learning (UCI) Datasets show that these improvements make MSNN outperform 187 neural and non-neural methods.

### Contribution of this work

(1) Revealing the root of the SNN association error to be the wrong state initialization method. (2) Updating SNN’s working process to solve the association error and release the parameter tuning space. (3) Proposing an EBP-based training method to enable adaptation of the built-in attention parameters to the data distribution.

## Related work

In general, classification methods for imbalanced data include data preprocessing, training target modification, and proposing targeting methods^35^. Since most classification networks are not designed with data imbalance, related research focuses on data preprocessing methods. Depending on the distributional characteristics of the data, preprocessing methods can be categorized into oversampling^36,37^, undersampling^38,39^, and hybrid methods of the two^40,41^. In recent years, with the increasing demand for data volume of classification networks and the proposal of pattern generation methods based on generative adversarial networks, oversampling of minority classes has gradually become a mainstream method^42,43^. However, our solution belongs to the category of proposing targeting methods, and our network natively supports imbalanced data for training, which is divergent from the above studies.

## SNN overview

### SNN’s working procedure

**Figure 1:**
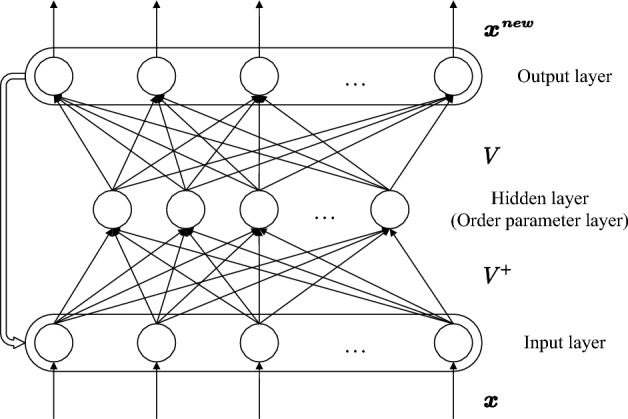
SNN structure. The activation function of the hidden layer is $Syn$. The input and output layers are linear mappings with weights of the adjoint matrix ${V}^{+}$ and the prototype matrix $V$, respectively.

SNN is a 3-layer RNN, its network structure is shown in Fig. 1. Updating formula of SNN^11^ of its input, hidden, and output layer is

1 $${\varvec{\xi}}={V}^{+}{\varvec{x}}$$

2 $${{\varvec{\xi}}}^{{\varvec{n}}{\varvec{e}}{\varvec{w}}}=Syn\left({\varvec{\xi}}\right)=\gamma \left(\frac{b{{\varvec{\xi}}}^{3}+{\varvec{\lambda}}{\varvec{\xi}}}{(b+c){\Vert {\varvec{\xi}}\Vert }_{2}^{2}}+\left(\frac{1}{\gamma }-1\right){\varvec{\xi}}\right)$$

3 $${{\varvec{x}}}^{{\varvec{n}}{\varvec{e}}{\varvec{w}}}=V{{\varvec{\xi}}}^{{\varvec{n}}{\varvec{e}}{\varvec{w}}}$$

${\varvec{x}}$ is the normalized query pattern. $V=[{{\varvec{v}}}_{1},\dots ,{{\varvec{v}}}_{{\varvec{M}}}]$ is the matrix of normalized static prototypes representing memories. ${{\varvec{x}}}^{{\varvec{n}}{\varvec{e}}{\varvec{w}}}$ is the new input transmitting to Eq. (1). ${\varvec{\xi}}$ is the vector of order parameters. ${V}^{+}$ is $V$’s Moore–Penrose inverse^44,45^. $Syn$ is the Synergetic activation function. $\gamma$ is the learning rate. Network parameters include ${\varvec{\lambda}}$, $b$, and $c$. ${\varvec{\lambda}}$ is the attention parameter to the prototypes with default value 1. Higher attention brings greater chances of association. $b$ and $c$ control the convergence speed with default value 1. SNN requires that all prototypes are mutually independent, and that their total number is less than the dimension, such that the product of ${V}^{+}$ and $V$ is the identity matrix. Substitute Eq. (3) into (1), and ${\varvec{\xi}}={V}^{+}{{\varvec{x}}}^{{\varvec{n}}{\varvec{e}}{\varvec{w}}}={{\varvec{\xi}}}^{{\varvec{n}}{\varvec{e}}{\varvec{w}}}$. Thus, the update formula can be interpreted as constructing a dynamic system of ${\varvec{\xi}}$. ${\varvec{\xi}}$ is the dynamical state, and its initial value is the initial state. The variation of ${\varvec{\xi}}$ is reflected to ${\varvec{x}}$ through $V$.

**Figure 2:**
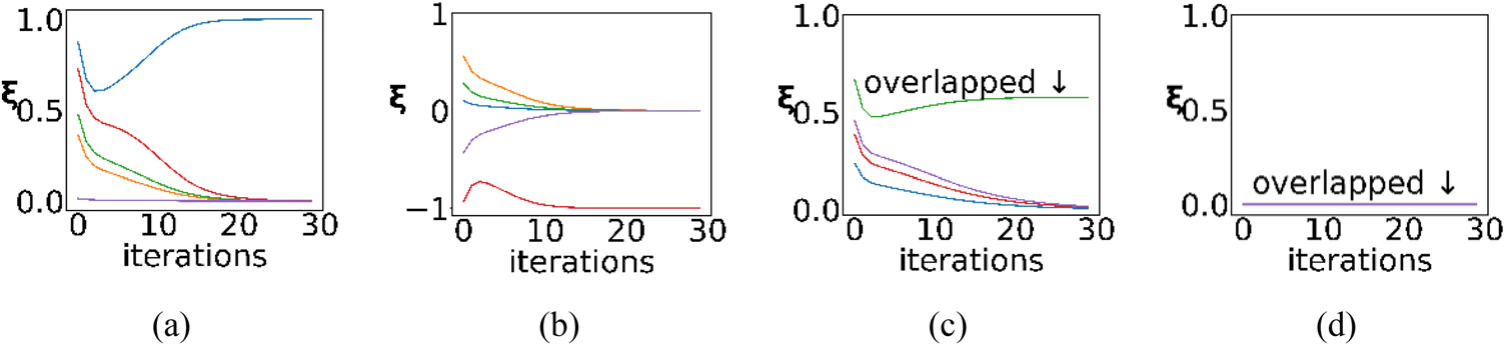
The convergence to stationary points of SNN. The different colors of the curves are used to distinguish order parameters. (**a**,**b**) are the convergence to target stable points (the positive or negative one-hot), i.e., one order parameter converges to ± 1 while others converge to 0; (**c**) is the convergence to the saddle point (multiple identical values) that stems from multiple identical extreme values in the initial value of ${\varvec{\xi}}$; (**d**) is the convergence to the local maximum point (all values are zeros) that stems from the zero-vector initialization of ${\varvec{\xi}}$. Note that chart (**d**) is only for representation. The divide-by-0 error terminates the network’s further iterations.

SNN converges to three kinds of stationary points, including the target stable point, the saddle point, and the local maxima point. The convergences are shown in Fig. 2. Generally, SNN reaches the target stable point. The target stable point is reached when ${\varvec{\xi}}$ is the positive or negative one-hot encoding. The single nonzero order parameter is called the winner parameter. The network outputs $\pm {\varvec{v}}$ at this point, which reflects the association from ${\varvec{x}}$ to ${\varvec{v}}$. The saddle point is reached when ${\varvec{\xi}}$ has more than one identical nonzero value, which stems from multiple identical extremes in the initial state. The local maximum point is reached when all elements of ${\varvec{\xi}}$ are 0. The division by 0 error in Eq. (2) blocks the network from working.

### SNN’s basin of attraction

**Figure 3:**
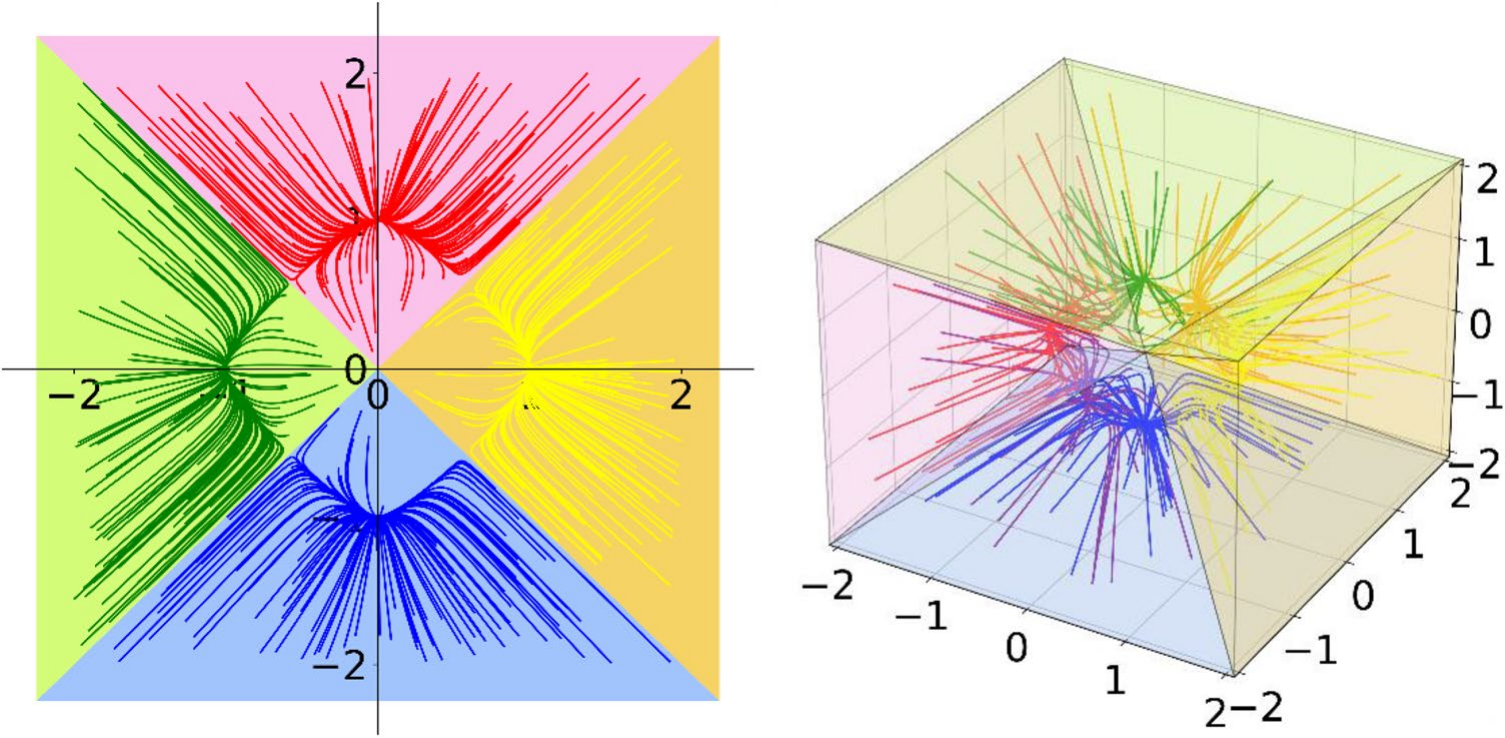
SNN’s attractors, basins, and trajectories of random initial states in 2D and 3D space. One coordinate of the attractor is ± 1 and others are 0. The trajectories and the basin corresponding to the same attractor are marked with the same type of color. The basin contains all initial states with the same sign and maximum absolute value index as its attractor.

In describing the convergence process to the target stabilization point, SNN proposes the “winner-takes-all” property, i.e., the order parameter with the biggest absolute initial value is the winner parameter, but lacking detailed proof. Therefore, we prove this property by showing that $\left|{\xi }_{m}^{new}\right|$ is the largest when $\left|{\xi }_{m}\right|$ is the largest. The detailed proof is shown in SI ***1A. From the perspective of dynamical systems, the “winner-takes-all” property can be interpreted as extreme-based basin partitioning. The basin of SNN’s attractor is the set of all initial states with the same sign and extreme value index as itself. The attractors, basins, and trajectories of random initial states of SNN in 2D and 3D are shown in Fig. 3. It can be seen that such a division allows the order parameter with the biggest absolute value retains its winner position throughout the convergence.

## MSNN

The nonlinear dynamical system is sensitive to the initial state and should be carefully designed. However, the initialization method of SNN was proposed without in-depth analysis. Although the associative memory task requires the correct association to be the most similar memory to the input, we prove that the existing initialization method will designate the order parameter with smaller similarity as the winner parameter. Due to the “winner-takes-all” property of SNN’s convergence, the selected winner will converge to ± 1, so the network will output the less similar memory as the association result, leading to the association error. To address this problem, we redesign the state initialization method to correct the winner designation process. The new approach ensures the consistency of the winner selection and the association target, fundamentally solving the association error problem of SNN. In addition, the new initialization method provides the feasibility of EBP-based parameter learning.

### SNN’s erroneous state initialization method

The working target of SNN is to converge to the most similar memory. The initial state controls the convergence, so the initialization method should be proposed under a similarity metric. However, the similarity between the sample ${\varvec{x}}$ and the memory ${\varvec{v}}$ cannot be characterized by the metric of SNN’s state initialization method

4 $$S\left({{\varvec{v}}}_{{\varvec{m}}},{\varvec{x}}\right)={{\varvec{v}}}_{{\varvec{m}}}^{+}\cdot {\varvec{x}}$$

Although there are at least 67 different metrics applied in various fields^46^, all similarity metrics shall satisfy the following three principles^47^:

1. Commonality related. The more commonality they share, the more similar they are.
2. Difference related. The more differences they have, the less similar they are.
3. The maximum is reached when identical.

However, $S$ actually characterizes the scaled cosine distance of ${{\varvec{v}}}_{{\varvec{m}}}^{+}$ and ${\varvec{x}}$, conforming to none of the above principles. For Principle (3), $S=1$ when ${\varvec{x}}={{\varvec{v}}}_{{\varvec{m}}}^{+}$, while $S={\Vert {{\varvec{v}}}_{{\varvec{m}}}^{+}\Vert }_{2}$ when ${\varvec{x}}={{\varvec{v}}}_{{\varvec{m}}}^{+}/{\Vert {{\varvec{v}}}_{{\varvec{m}}}^{+}\Vert }_{2}$ (i.e., ${\varvec{x}}$ is the normalized adjoint vector). From "Related work", ${V}^{+}V$ is the identity matrix, so

5 $${{\varvec{v}}}_{{\varvec{m}}}^{+}\cdot {{\varvec{v}}}_{{\varvec{n}}}=\left\{\begin{array}{l}1,m=n\\ 0,otherwise\end{array}\right.$$

which means that ${{\varvec{v}}}_{{\varvec{m}}}^{+}$ is perpendicular to the hyperplane of all prototypes except ${{\varvec{v}}}_{{\varvec{m}}}$. Since the inner product of ${{\varvec{v}}}_{{\varvec{m}}}^{+}$ and ${{\varvec{v}}}_{{\varvec{m}}}$ is 1, the angle between ${{\varvec{v}}}_{{\varvec{i}}}^{+}$ and ${{\varvec{v}}}_{{\varvec{m}}}$ takes values in the range [0,0.5π). SNN requires ${{\varvec{v}}}_{{\varvec{m}}}^{+}$ to be normalized, so ${\Vert {{\varvec{v}}}_{{\varvec{m}}}^{+}\Vert }_{2}\ge 1$. $S$ may achieve a bigger value when it is not equal to ${{\varvec{v}}}_{{\varvec{m}}}$, so $S$ does not satisfy Principle (3). For Principle (1) and (2), as ${\varvec{x}}$ gradually approaches ${{\varvec{v}}}_{{\varvec{m}}}^{+}$ from ${{\varvec{v}}}_{{\varvec{i}}}$, its commonality with ${{\varvec{v}}}_{{\varvec{m}}}$ decreases and the difference increases, but $S$ increases other than decreases. Therefore, $S$ does not satisfy Principles (1) and (2).

The conflict between $S$ and the similarity metric causes the association error. From the previous section, the order parameter with the largest absolute value in the initial state is the winner parameter. SNN will pick the wrong winner when the largest order parameter relates to a less similar ${\varvec{v}}$ by $S$, which leads to an association error.

### MSNN’s remodeling of the state initialization method

The association error originates from the wrong initial state, so the MSNN needs to redesign the initialization method. Since SNN’s basin of attraction focuses on the parameter’s absolute value, simply using the similarity measure as the state initialization method of SNN may allow the smallest negative order parameter to be the winner, making the network associates the least similar memory. To avoid this problem, we propose the new initialization method as

6 $${\xi }_{m}=ReLU\left(S\left({\varvec{x}},{{\varvec{v}}}_{{\varvec{m}}}\right)\right)$$

$S$ is the similarity measure between the query and the memory. $ReLU$ sets the negative value to zero, eliminating the possibility of the negative order parameter becoming the winner. In summary, the working process of MSNN is

7 $${\varvec{\xi}}=\left\{\begin{array}{c}ReLU\left({\left(S\left({\varvec{x}},{{\varvec{v}}}_{1}\right),\dots ,S\left({\varvec{x}},{{\varvec{v}}}_{{\varvec{M}}}\right)\right)}^{\mathrm{T}}\right),\xi \ not \ initialized\\ {V}^{+}x, otherwise\end{array}\right.$$

8 $${{\varvec{\xi}}}^{{\varvec{n}}{\varvec{e}}{\varvec{w}}}=Syn\left({\varvec{\xi}}\right)$$

9 $${{\varvec{x}}}^{{\varvec{n}}{\varvec{e}}{\varvec{w}}}=V{{\varvec{\xi}}}^{{\varvec{n}}{\varvec{e}}{\varvec{w}}}$$

**Figure 4:**
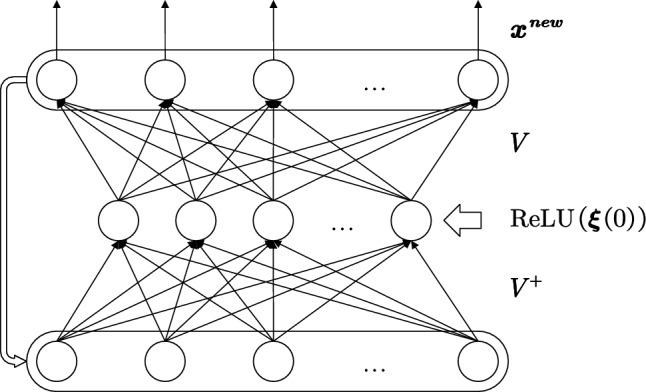
The working process of the MSNN after the correction of the state initialization method. The network input ${\varvec{x}}$ is mapped to ${\varvec{\xi}}\left(0\right)$ through the corrected state initialization, ${\varvec{\xi}}\left(0\right)$ is activated by the ReLU function and input to the hidden layer by the initial order parameter feedforward layer, and then the network starts to work in iterations.

MSNN’s network structure is shown in Fig. 4.

The new initialization method ensures the correct association while improving the running speed. This method only allows the positive value to be the initial value of the order parameter, so the most similar memory must correspond to the largest order parameter. From the “winner-takes-all” property, the largest order parameter becomes the winner, and the most similar memory becomes the association result. The new initialization method sparsifies ${\varvec{\xi}}$ by setting negative order parameters to zero, thus speeding up the hardware computation.

### MSNN’s attention parameter self-learning

SNN’s genetic algorithm-based parameter learning is hard to be co-trained with other modern network layers, so we design an EBP-based learning method. The new learning method adjusts the attention parameter ${\varvec{\lambda}}$ to assign greater attention to classes with smaller sizes for imbalanced data self-adaptation. Before applying EBP, $Syn$ repeatedly imposes a polynomial function onto the input, which may lead to the gradient exploding or vanishing. The gradient problem is so severe that conventional means like gradient clipping can barely circumvent the non-convergence. To solve this problem, we first normalize ${\varvec{\xi}}$ and divide $Syn$ into two terms,

10 $$Syn\left({\varvec{\xi}}\right)=\frac{\gamma{\varvec{\lambda}}{\varvec{\xi}}}{b+c}+\left(\frac{\gamma b{{\varvec{\xi}}}^{3}}{b+c}+\left(1-\gamma \right){\varvec{\xi}}\right)$$

EBP is performed normally for the former term, and the latter term uses the gradient bypass technique^48,49^. This technique passes the gradient of certain network layer outputs directly to the input during backpropagation, which is used to circumvent the inappropriate activation functions causing the gradient exploding or vanishing, even the gradient intransmissible caused by discontinuity.

The parameter learning requirement can be satisfied by directly acting EBP onto ${\varvec{\lambda}}$. Let the error of ${\xi }_{i}^{new}$ be ${\delta }_{i}$. ${\xi }_{i}\ge 0$,

11 $$\frac{\partial {\delta }_{i}}{\partial {\lambda }_{i}}=\frac{\gamma {\xi }_{i}}{b+c}\ge 0$$

so the adjustment $\Delta {\lambda }_{i}$ has a different sign than ${\delta }_{i}$. ${\delta }_{i}>0$ means ${\xi }_{i}^{new}$ is too large, and $\Delta {\lambda }_{i}\le 0$ means the network will not increase its attention to ${\xi }_{i}$, giving it a higher chance to converge to 0. ${\delta }_{i}<0$ means ${\xi }_{i}^{new}$ is too small, and $\Delta {\lambda }_{i}\ge 0$ leads ${\xi }_{i}$ to a higher chance of converging to 1. Therefore, EBP satisfies the parameter learning requirement of ${\varvec{\lambda}}$.

## Experiments

### Dataset and network configuration

We test MSNN on the small datasets of the UCI, a collection of 121 datasets as pattern classification tasks to benchmark both neural network and non-neural network machine learning algorithms. These datasets are divided into 75 small and 46 large datasets by the threshold of 1000 samples^50^. All of these datasets are imbalanced after the train-test set division. We compare our network against 187 neural and non-neural machine learning algorithms. Their configurations and performances are detailed in literatures^16,17,50^. See SI 1B.2 for dataset configuration details.

**Figure 5:**
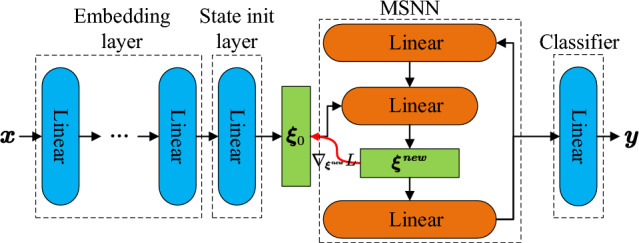
Network structure for experiments. The network input ${\varvec{x}}$ is preprocessed through the embedding layer and subsequently transformed into the initial state of the SNN through the initialization layer, i.e., the initial value of the ordinal parameter ${{\varvec{\xi}}}_{0}$. ${{\varvec{\xi}}}_{0}$ is passed into the hidden layer of the SNN, and the update of the ordinal parameter and the association of memory are realized with the cycle of the network. The prototype pattern obtained by association is passed into the classifier by the output layer of the SNN to obtain the label y. The parameters are trained using error backpropagation with the loss function $CrossEntropy\left({\varvec{y}},{\varvec{l}}{\varvec{a}}{\varvec{b}}{\varvec{e}}{\varvec{l}}\right)$, where ${\varvec{l}}{\varvec{a}}{\varvec{b}}{\varvec{e}}{\varvec{l}}$ is the true label of the data. Using the gradient direct transmission technique, the gradient ${\nabla }_{\varvec{\xi^{new}}}\ L$ of ${{\varvec{\xi}}}^{{\varvec{n}}{\varvec{e}}{\varvec{w}}}$ in the error backpropagation stage (red line in the figure) is passed directly to ${{\varvec{\xi}}}_{0}$ bypassing the polynomial-shaped activation function of the SNN to circumvent gradient explosion or vanish.

As for the network architecture, we use the embedding layers, which is/are {0, 1, 7} fully connected layer(s) with ReLU activation functions and {32, 128, 1024} hidden units per embedding layer. These embedding layers are followed by SNN with iteration {0} to {9} and a mapping to the output vector with the dimension number of classes. The prototype matrix is obtained by intra-class K-means clustering, and the adjoint matrix is the M-P inverse of the prototype matrix. The network structure used for the experiments is shown in Fig. 5. On each dataset, we use EBP to train SNN’s hyperparameter ${\varvec{\lambda}}$ and perform a grid search to determine the best hyperparameter setting for the embedding layers, the memory number, and SNN’s iteration number. The hyperparameter search space of the grid search is listed in Table 1. All models are trained for 100 epochs with a mini-batch size of 4 samples using the softmax cross-entropy loss and the AdamW optimizer^51^. After each epoch, the model accuracy is computed on a separate validation set. Using the gradient direct transmission technique^48,49^, the gradient of MSNN’s output layer in the error backpropagation stage is passed directly to the state initialization layer to circumvent bypassing the polynomial-shaped activation function of the SNN causing gradient exploding or vanishing. With early stopping, the model with the best validation set accuracy averaged over 16 consecutive epochs is selected as the final model. This final model is then evaluated against the test set to determine the accuracy.

**Table 1:** Hyperparameter search space for grid search on small UCI datasets.

Parameter	Values
Embedding layers	{0,1,7}
Hidden units	{32,128,1024}
Stored patterns	{1,8}·*class_num*
SNN iteration	{0,1,2,3,4,5,6,7,8,9}

All models are trained for 100 epochs with a minibatch size of 4 using AdamW with early stopping based on the accuracy. The number of stored patterns is 1 or 8 times the number of the target classes of the individual task.

### Classification performances validation

The Friedman rankings of these methods among datasets are presented in Table 2. MSNN outperforms all other methods on small datasets, setting a new state-of-the-art for 12 datasets (balance-scale, breast-cancer, congressional-voting, heart-cleveland, ionosphere, low-res-spect, monks-2, monks-3, planning, post-operative, soybean, and spect). See SI 1B ***for more details.

**Table 2:** Friedman ranking and average accuracy (%) for each classifier, ordered by increasing Friedman ranking.

Classifier	Rank	Acc
**MSNN (proposed)**	**30.00**	**83.40**
M_hopfield	32.24	82.64
svmPoly_caret	33.39	79.79
rf_caret	38.43	80.28
avNNet_caret	38.73	79.00
svmRadialCost_caret	38.85	79.82
elm_kernel_matlab	39.83	80.52
pcaNNet_caret	39.88	79.64
…	…	…
ClassificationViaClustering_weka	161.92	54.27

Significant values are in [bold].

### Imbalanced data adaptation performance

**Figure 6:**
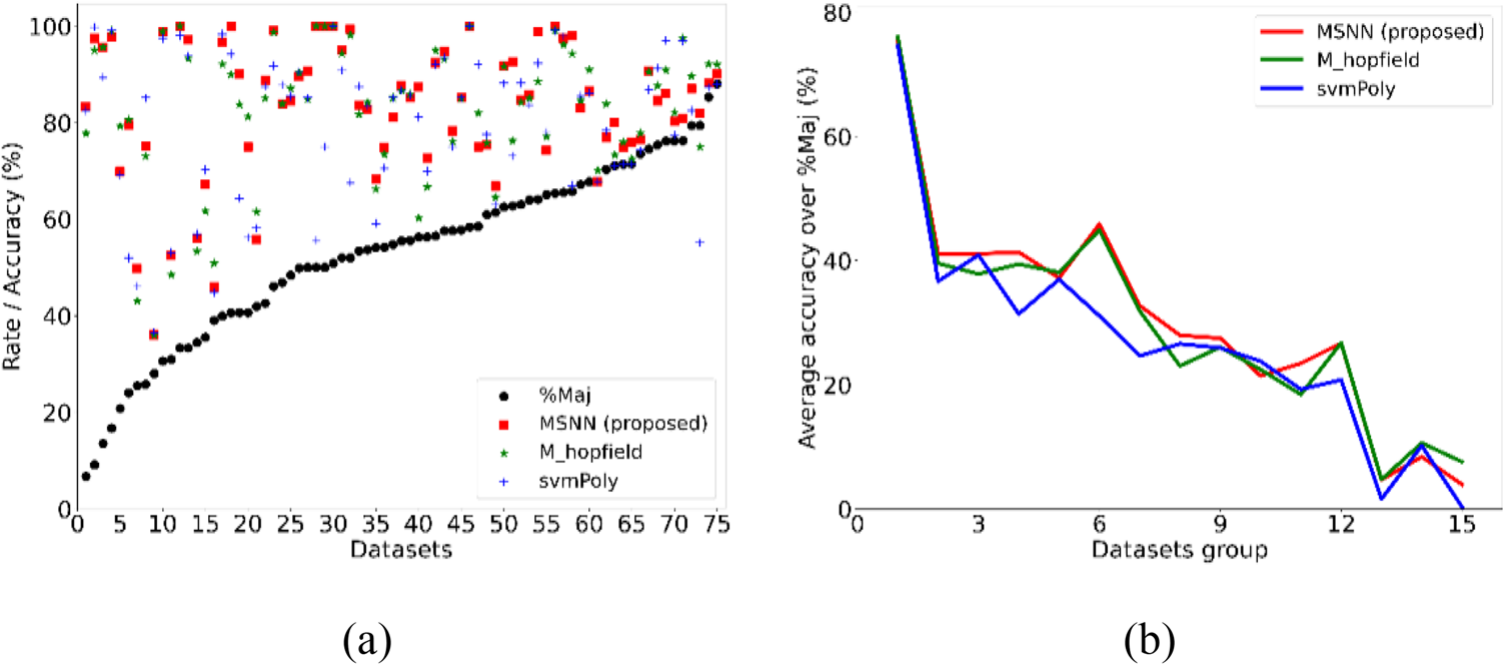
(**a**) Majority class percentage %Maj of small UCI datasets and the accuracy of Friedman ranking top three methods. Datasets are ranked in ascending order of %Maj. (**b**) The average accuracy over %Maj (average $\sigma$) of the three methods with the adjacent datasets merged in the group of five. MSNN has the highest average $\sigma$ in the first 12 groups except group 10, with a decrease in groups 13–15, indicating its better performance for mild and moderate imbalance datasets.

We analyze the performance of MSNN for datasets with different levels of imbalance in terms of the percentage of majority class %Maj^17^. %Maj reflects the level of imbalance in the dataset, the higher the %Maj, the higher the imbalance. The classifier is prone to focus on the majority class when applied to imbalanced data, i.e., labeling all samples as the major class, which brings the overfitting problem. The more severe the overfitting problem is, the closer the accuracy of the classifier will be to %Maj. Thus, the accuracy over %Maj, denoted by $\sigma$, can reflect the extent of minor class samples being correctly classified. See SI 1B ***for the %Maj of each UCI dataset. We rank each dataset in ascending order of %Maj and mark the accuracies of the top three Friedman ranking methods in Fig. 6a. We merge the adjacent datasets in groups of five and calculate their average $\sigma$ for better visualization. The results are shown in Fig. 6b. MSNN outperforms other methods in most cases, and the average $\sigma$ improvement is most obvious in groups 2–12 (except group 10) with the %Maj interval of (30.93, 67.83), which indicates that MSNN is useful for both mild and moderate imbalance datasets have good adaptive performance. In groups 13–15 with %Maj greater than 73.53, the average $\sigma$ of MSNN has a decrease compared to other methods, which suggests that the linear classifier and the standard associative memory network have a more stable performance for heavily imbalanced datasets.

### Order parameter initialization validation

**Figure 7:**
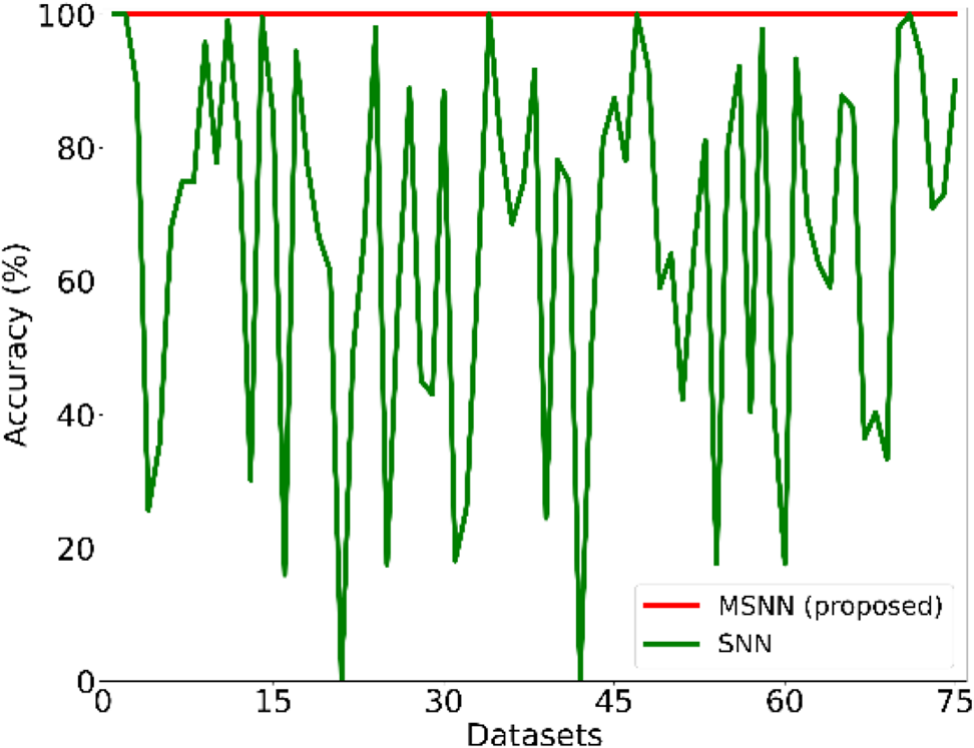
The associative accuracy of SNN and MSNN. The datasets are ordered by name. MSNN reaches 100% accuracy in all datasets, while SNN’s performance fluctuates significantly and reaches 100% accuracy in only 5 datasets.

We verify the effectiveness of MSNN’s order parameter initialization method for correcting association errors by comparing the accuracy to SNN. We use the balanced parameter configuration (all parameters default to be 1), so the association target is the most similar memory to the query. The performance of the SNN and MSNN is shown in Fig. 7. MSNN achieves 100% accuracy for all datasets, while SNN achieves 100% accuracy in only 5 datasets (acute-inflammation, acute-nephritis, horse-colic, monks-3, and trains). Its average accuracy is 66.47%.

### Attention parameter learning performance

MSNN mitigates overfitting by attention parameter ${\varvec{\lambda}}$ self-learning to provide greater attention to classes with small sample sizes. Ideally, the elements of ${\varvec{\lambda}}$ should negatively correlate with the number of imbalanced samples. Due to the diversity of data sources, MSNN cannot guarantee fitting effectiveness on all datasets, and the attention parameter’s learning performance can hardly be reflected in the underperformed datasets. In addition, the correlation between ${\varvec{\lambda}}$ and the number of imbalanced samples is challenging to model in a parameter configuration with multiple attention parameters corresponding to one class. Since the objective is to verify the ideal cases rather than all cases, we dropped results from 28 datasets that did not meet the criteria, including (1) positive effect of ${\varvec{\lambda}}$ self-learning on performance. (2) The number of ${\varvec{\lambda}}$ is equal to the class number. We use Spearman correlation analysis to verify the correlation between ${\varvec{\lambda}}$ and the sample sizes. Spearman analysis requires at least four samples, yet a significant proportion of 2 or 3 class tasks are in the UCI dataset. Therefore, we apply 1-norm to ${\varvec{\lambda}}$ from different datasets and integrate them. The integrated ${\varvec{\lambda}}$ contains 192 samples with a correlation coefficient of about − 0.170, corresponding to a *p* value of about 0.019. Thus, ${\varvec{\lambda}}$ has a significantly negative correlation with the sample sizes, indicating that EBP is applicable to the learning of ${\varvec{\lambda}}$.

## Conclusion

In this paper, we propose the MSNN model to further improve RNN’s classification performance on imbalanced small data. MSNN first addresses SNN’s misattributing association errors to underoptimized parameters in existing studies by modifying the state initialization method in its working process, releasing the whole parameter optimization space to task requirements. Then, MSNN adjusts SNN’s built-in attention parameter through an EBP and error bypass-based learning method for network self-adaptation of imbalanced data during network layers joint training. Experimental results on 75 small UCI datasets show that MSNN retains error-free associations on all datasets, and the attention parameters spontaneously establish a strong correlation with the imbalanced sample size. These improvements make MSNN outperforms 187 methods and achieves a new state-of-the-art.

Our study allows the theoretical advantages of the Synergetics to be successfully applied in artificial neural networks, and we plan to further extend these advantages to other areas in future work, including optimization methods for attention mechanisms and self-learning methods of representative prototypes.

### Supplementary Information

Supplementary Information 1.

Supplementary Information 2.

## Data Availability

Publicly available datasets were analyzed in this study. This data can be found here: https://archive.ics.uci.edu/datasets.

## References

[1] Ba J, Hinton G, Mnih V, Leibo JZ, Ionescu C (2016). Using fast weights to attend to the recent past. Adv. Neural Inf. Process. Syst..

[2] Wu X, Liu X, Li W, Wu Q (2018). Improved expressivity through dendritic neural networks. Adv. Neural. Inf. Process. Syst..

[3] Schlag I, Schmidhuber J (2018). Learning to reason with third-order tensor products. Adv. Neural. Inf. Process. Syst..

[4] Radhakrishnan A, Belkin M, Uhler C (2020). Overparameterized neural networks implement associative memory. Proc. Natl. Acad. Sci. USA.

[5] Huang, C., Li, Y., Loy, C. C. & Tang, X. Learning deep representation for imbalanced classification. In *Proceedings of the IEEE Computer Society Conference on Computer Vision and Pattern Recognition*, Vol. 2016, 5375–5384 (2016).

[6] Khan SH, Hayat M, Bennamoun M, Sohel FA, Togneri R (2018). Cost-sensitive learning of deep feature representations from imbalanced data. IEEE Trans. Neural Netw. Learn. Syst..

[7] Yan, Y., Chen, M., Shyu, M. L. & Chen, S. C. Deep Learning for Imbalanced Multimedia Data Classification. In *Proceedings—2015 IEEE International Symposium on Multimedia, ISM 2015*, 483–488 (2016). 10.1109/ISM.2015.126.

[8] Shorten C, Khoshgoftaar TM (2019). A survey on image data augmentation for deep learning. J. Big Data.

[9] Gao JIE (2020). Data augmentation in solving data imbalance problems. Degree Proj. Comput. Sci. Eng..

[10] Wen Q (2021). Time series data augmentation for deep learning: A survey. IJCAI Int. Jt. Conf. Artif. Intell..

[11] Haken H (1991). Synergetic Computers and Cognition : A Top-Down Approach to Neural Nets. Springer Series in Synergetics, ***Vol. 50.

[12] Hopfield JJ (1982). Neural networks and physical systems with emergent collective computational abilities. Proc. Natl. Acad. Sci. USA.

[13] Kosko B (1988). Bidirectional associative memories. IEEE Trans. Syst. Man Cybern..

[14] Adachi M, Aihara K (1997). Associative dynamics in a chaotic neural network. Neural Netw..

[15] Krotov D, Hopfield JJ (2016). Dense associative memory for pattern recognition. Advances in Neural Information Processing Systems.

[16] Ramsauer, H. *et al.* Hopfield Networks is All You Need. http://arxiv.org/abs/2008.02217 (2020).

[17] Fernández-Delgado M, Cernadas E, Barro S, Amorim D (2014). Do we need hundreds of classifiers to solve real world classification problems?. J. Mach. Learn. Res..

[18] Wang H, Yu Y, Wen G, Zhang S, Yu J (2015). Global stability analysis of fractional-order Hopfield neural networks with time delay. Neurocomputing.

[19] Wu A, Zeng Z, Song X (2016). Global Mittag–Leffler stabilization of fractional-order bidirectional associative memory neural networks. Neurocomputing.

[20] Yang Z, Zhang J (2020). Global stabilization of fractional-order bidirectional associative memory neural networks with mixed time delays via adaptive feedback control. Int. J. Comput. Math..

[21] Demircigil M, Heusel J, Löwe M, Upgang S, Vermet F (2017). On a model of associative memory with huge storage capacity. J. Stat. Phys..

[22] Barra A, Beccaria M, Fachechi A (2018). A new mechanical approach to handle generalized Hopfield neural networks. Neural Netw..

[23] Zhao T, Tang LH, Ip HHS, Qi F (2003). On relevance feedback and similarity measure for image retrieval with synergetic neural nets. Neurocomputing.

[24] Wong, W. M., Loo, C. K. & Tan, A. W. C. Parameter controlled chaotic synergetic neural network for face recognition. In *2010 IEEE Conference on Cybernetics and Intelligent Systems, CIS 2010*, 58–63 (2010). 10.1109/ICCIS.2010.5518581.

[25] Huang Z, Chen Y, Shi X (2012). A parallel SRL algorithm based on synergetic neural network. J. Converg. Inf. Technol..

[26] Huang Z, Chen Y, Shi X (2017). A synergetic semantic role labeling model with the introduction of fluctuating force accompanied with word sense information. Intell. Data Anal..

[27] Hu D, Qi F (1998). Reconstruction of order parameters in synergetics approach to pattern recognition. J. Infrared Millim. Waves.

[28] Ma X, Jiao L (2004). Reconstruction of order parameters based on immunity clonal strategy for image classification. Lect. Notes Comput. Sci..

[29] Ma X, Wang S, Jiao L (2005). Robust classification of immunity clonal synergetic network inspired by fuzzy integral. Lect. Notes Comput. Sci..

[30] Chen Y, Huang Z, Shi X (2016). An SNN-based semantic role labeling model with its network parameters optimized using an improved PSO algorithm. Neural Process. Lett..

[31] Gou SP, Jiao LC, Tian XL (2008). Image recognition using synergetic neural networks based on immune clonal clustering. J. Electron. Inf. Technol..

[32] Wagner T, Boebel FG (1994). Testing synergetic algorithms with industrial classification problems. Neural Netw..

[33] Li, H., Ma, X., Wan, W. & Zhou, X. Image similarity matching retrieval on synergetic neural network. In *ICALIP 2010–2010 International Conference on Audio, Language and Image Processing, Proceedings*, 1566–1571 (2010). 10.1109/ICALIP.2010.5684499.

[34] Liu S, Liu Z, Sun J, Liu L (2011). Application of synergetic neural network in online writeprint identification. Int. J. Digit. Content Technol. Appl..

[35] Kaur H, Pannu HS, Malhi AK (2019). A systematic review on imbalanced data challenges in machine learning: Applications and solutions. ACM Comput. Surv..

[36] Zheng Z, Cai Y, Li Y (2015). Oversampling method for imbalanced classification. Comput. Inform..

[37] Moreo, A., Esuli, A. & Sebastiani, F. Distributional random oversampling for imbalanced text classification. In *SIGIR 2016—Proceedings of the 39th International ACM SIGIR Conference on Research and Development in Information Retrieval*, 805–808 (2016). 10.1145/2911451.2914722.

[38] Yu H, Ni J, Zhao J (2013). ACOSampling: An ant colony optimization-based undersampling method for classifying imbalanced DNA microarray data. Neurocomputing.

[39] Dai, D. & Hua, S.-W. Random Under-sampling ensemble methods for highly imbalanced rare disease classification. In *12th International Conference on Data Mining*, 54–59 (2016).

[40] Qian Y, Liang Y, Li M, Feng G, Shi X (2014). A resampling ensemble algorithm for classification of imbalance problems. Neurocomputing.

[41] Charte F, Rivera AJ, del Jesus MJ, Herrera F (2015). Addressing imbalance in multilabel classification: Measures and random resampling algorithms. Neurocomputing.

[42] Nugraha RA, Pardede HF, Subekti A (2022). Oversampling based on generative adversarial networks to overcome imbalance data in predicting fraud insurance claim. Kuwait J. Sci..

[43] Fanny S, Cenggoro TW (2018). Deep learning for imbalance data classification using class expert generative adversarial network. Proced. Comput. Sci..

[44] Moore EH (1920). On the reciprocal of the general algebraic matrix. Bull. Am. Math. Soc..

[45] Penrose R (1955). A generalized inverse for matrices. Math. Proc. Camb. Philos. Soc..

[46] McGill M, Koll MTN (1979). An evaluation of factors affecting document ranking by information retrieval systems. Algorithms.

[47] Lin D (1998). An information-theoretic definition of similarity. Icml.

[48] Van Den Oord A, Vinyals O, Kavukcuoglu K (2017). Neural discrete representation learning. Adv. Neural. Inf. Process. Syst..

[49] Razavi A, van den Oord A, Vinyals O (2019). Generating diverse high-fidelity images with VQ-VAE-2. Adv. Neural. Inf. Process. Syst..

[50] Klambauer G, Unterthiner T, Mayr A, Hochreiter S (2017). Self-normalizing neural networks. Adv. Neural. Inf. Process. Syst..

[51] Loshchilov, I. & Hutter, F. *Fixing Weight Decay Regularization in Adam*. *Iclr* https://openreview.net/pdf?id=Bkg6RiCqY7 (2018).

