# Supplementary material for: Modern synergetic neural network for imbalanced small data classification
Source: Sci Rep. 2023 Sep 21;13:15669. doi: 10.1038/s41598-023-42689-8 (PMC10514188; doi:10.1038/s41598-023-42689-8)
Supplement: Supplementary file 1 — Supplementary Information 1. [file 41598_2023_42689_MOESM1_ESM.docx]

**Appendix A. Proof of the “Winner-takes-all” Property**

We prove that $\left| \xi_{m}^{new} \right|$ is the largest when $\left| \xi_{m} \right|$. Let $\xi_{m}>0$ and

$$\begin{aligned} g\left( \xi_{m} \right)=\xi_{m}^{new}-\xi_{m}=\gamma\left( \frac{\xi_{m}^{3}+\xi_{m}}{2\left\| \boldsymbol{\xi} \right\|_{2}^{2}}-\xi_{m} \right)\#\left( A.1 \right) \end{aligned}$$

Let $k=1-2\left\| \boldsymbol{\xi} \right\|_{2}^{2}$,

$$\begin{aligned} g\left( \xi_{m} \right)=\frac{\gamma}{2\left\| \boldsymbol{\xi} \right\|_{2}^{2}}\left( \xi_{m}^{3}+k\xi_{m} \right)\#\left( A.2 \right) \end{aligned}$$

Discuss $k$:

1. $k\geq0$

$g\left( \xi_{m} \right)$is a monotonically increasing odd function, so $\xi_{m}$ has maximum increment during the update.

2. $k<0$

$g\left( \xi_{m} \right)$ has two stationary points $\pm\sqrt{\left( -k \right)/3}$ and two zero points $\pm\sqrt{-k}$ besides the origin. We construct constant $\alpha>\sqrt{-k}$ that satisfies $g\left( \alpha\right)=g\left( -\sqrt{\left( -k \right)/3} \right)$ and discuss $\xi_{m}$:

(1) $\xi_{m}>\alpha$

$g\left( \xi_{m} \right)>0$ and $g\left( \xi_{m} \right)>g\left( \xi_{m^{'}} \right)$, so $\xi_{m}$ has maximum increment during the update.

(2) $\sqrt{-k}<\xi_{m}\leq\alpha$

Construct two constants $-\sqrt{-k}<\beta_{1}<\beta_{2}<0$ that satisfy $g\left( \beta_{1} \right)=g\left( \beta_{2} \right)=g\left( \xi_{m} \right)$.$g\left( \xi_{m} \right)>0$ and $g\left( \xi_{m} \right)>g\left( \xi_{m^{'}} \right)$ when $\xi_{m^{'}}\notin\left( \beta_{1},\beta_{2} \right)$, so $\xi_{m}$ has maximum increment during the update. Although $g\left( \xi_{m} \right)<g\left( \xi_{m^{'}} \right)$ when $\xi_{m^{'}}\in\left( \beta_{1},\beta_{2} \right)$, $\xi_{m^{'}}$and $g\left( \xi_{m^{'}} \right)$ have different signs, $\xi_{m}$ increases and $\xi_{m^{'}}$ decreases, so $\xi_{m}$ has maximum increment during the update.

(3) $0<\xi_{m}\leq\sqrt{-k}$

Construct one constant $0<\beta_{3}<\sqrt{\left( -k \right)/3}$ that satisfies $f\left( \beta_{3} \right)=f\left( \xi_{m} \right)$.$g\left( \xi_{m^{'}} \right)<g\left( \xi_{m} \right)<0$ when $\xi_{m^{'}}\in\left( \beta_{3},\xi_{m} \right)$, so $\xi_{m}$ has minimum decrement during the update. Suppose there exists $\xi_{m^{'}}$ s.t. $g\left( \xi_{m} \right)<g\left( \xi_{m^{'}} \right)<0$ when $\xi_{m^{'}}\in\left( 0,\beta_{3} \right)$. Substitute equation (A.2) into the first two terms of the inequality and then shift the terms,

$$\begin{aligned} \xi_{m}-\xi_{m^{'}}<-\frac{\gamma}{2\left\| \boldsymbol{\xi} \right\|_{2}^{2}}\left( \left( \xi_{m}^{3}-\xi_{m^{'}}^{3} \right)+k\left( \xi_{m}-\xi_{m^{'}} \right) \right)\#\left( A.3 \right) \end{aligned}$$

Divide $\xi_{m}-\xi_{m^{'}}$ on both sides,

$$\begin{aligned} 1<-\frac{\gamma}{2\left\| \boldsymbol{\xi} \right\|_{2}^{2}}\left( \left( \xi_{m}^{2}+\xi_{m^{'}}^{2}+\xi_{m}\xi_{m^{'}} \right)+k \right)\#\left( A.4 \right) \end{aligned}$$

thus

$$\begin{aligned} \xi_{m}^{2}+\xi_{m^{'}}^{2}+\xi_{m}\xi_{m^{'}}<-k-\frac{2\left\| \boldsymbol{\xi} \right\|_{2}^{2}}{\gamma}\#\left( A.5 \right) \end{aligned}$$

Substitute $k=1-2\left\| \boldsymbol{\xi} \right\|_{2}^{2}$ to the right term,

$$\begin{aligned} -k-\frac{2\left\| \boldsymbol{\xi} \right\|_{2}^{2}}{\gamma}=(2-2/\gamma)\left\| \boldsymbol{\xi} \right\|_{2}^{2}-1\#\left( A.6 \right) \end{aligned}$$

The above formula is smaller than 0 when $\gamma\leq1$. $\xi_{m}$ and$\xi_{m^{'}}$ are both greater than 0, so the left side of (A.5) is also greater than 0, and (A.5) does not hold. Therefore, no $\xi_{m^{'}}$ satisfies $g\left( \xi_{m} \right)<g\left( \xi_{m^{'}} \right)<0$ when $\gamma\leq1$, $\xi_{m}$ has minimum decrement during the update.

In summary, $\xi_{m}$ has maximum increment or minimum decrement during the update when it is greater than 0. Following the step above, it is easy to prove that $\xi_{m}$ has minimum increment or maximum decrement during the update when it is smaller than 0. Therefore, $\xi_{m}$ with the largest absolute value remains largest.

**Appendix B. Details of the Experiments on Small UCI Datasets**

**B.1 Algorithms compared.** Our method is compared to 17 groups of methods ^10,11,35^:

1. Support Vector Machines

2. Random Forest

3. Multivariate adaptive regression splines (MARS)

4. Boosting

5. Rule-based Methods

6. Logistic and Multinomial Regression (LMR)

7. Discriminant Analysis (DA)

8. Bagging

9. Nearest Neighbor

10. Decision Trees

11. Other Ensembles

12. Neural Networks (Hopfield NN, standard NN, BatchNorm, WeightNorm, MSRAinit, LayerNorm, ResNet, Self-Normalizing Nets)

13. Bayesian Methods

14. Other Methods

15. Generalized linear models (GLM)

16. Partial Least Squares and Principal Component Regression (PLSR)

17. Stacking (Wolpert)

**B.2 Experimental design and implementation details.** We consider 75 small UCI datasets ^35^ containing less than 1,000 samples per dataset. The details of these datasets, including their name, data size, data dimension (Dim.), class number, and majority class rate (%Maj), are listed in Table B1. The train-test set separation is from paper ^11^. We compare the performance of the algorithms based on their average rank over all 75 datasets, as reported in Table 2 in the main text and detailed in Table B2. Classification accuracies of all methods are listed in *uci_results.pdf*.

Table B1. Collection of 75 small UCI datasets with their name, data size, data dimension (Dim.), class number, and majority class rate (%Maj).

| **Name** | **Size** | **Dim.** | **Class** | **%Maj (%)** | **Name** | **Size** | **Dim.** | **Class** | **%Maj (%)** |
| --- | --- | --- | --- | --- | --- | --- | --- | --- | --- |
| acute-inflam. | 120 | 6 | 2 | 50.83 | libras | 360 | 90 | 15 | 6.67 |
| acute-nephritis | 120 | 6 | 2 | 58.33 | low-res-spect | 531 | 100 | 9 | 51.98 |
| annealing | 898 | 38 | 5 | 76.17 | lung-cancer | 32 | 56 | 3 | 40.63 |
| arrhythmia | 452 | 279 | 13 | 54.20 | lymphography | 148 | 18 | 4 | 54.73 |
| audiology-std | 196 | 69 | 18 | 23.98 | mammographic | 961 | 5 | 2 | 53.69 |
| balance-scale | 625 | 4 | 3 | 46.08 | molec-biol-promoter | 106 | 57 | 2 | 50.00 |
| balloons | 16 | 4 | 2 | 56.25 | monks-1 | 556 | 6 | 2 | 50.00 |
| blood | 748 | 5 | 2 | 76.20 | monks-2 | 601 | 6 | 2 | 65.72 |
| breast-cancer | 569 | 30 | 2 | 62.74 | monks-3 | 554 | 6 | 2 | 51.99 |
| breast-cancer-w. | 198 | 33 | 2 | 76.26 | musk-1 | 476 | 166 | 2 | 56.51 |
| breast-cancer-w.-d. | 699 | 9 | 2 | 65.52 | oocytes_t._n. | 912 | 25 | 2 | 57.79 |
| breast-cancer-w.-p. | 286 | 9 | 2 | 70.28 | oocytes_t._s. | 912 | 32 | 3 | 57.57 |
| breast-tissue | 106 | 9 | 6 | 20.75 | parkinsons | 195 | 23 | 2 | 75.38 |
| congressional-v. | 435 | 16 | 2 | 61.38 | pima | 768 | 8 | 2 | 65.10 |
| conn-bench-s. | 208 | 60 | 2 | 53.37 | pittsburg-b.-M. | 106 | 7 | 3 | 74.53 |
| conn-bench-v. | 990 | 11 | 11 | 9.09 | pittsburg-b.-R. | 103 | 7 | 3 | 56.31 |
| credit-approval | 690 | 15 | 2 | 55.51 | pittsburg-b.-S. | 92 | 7 | 3 | 57.61 |
| cylinder-bands | 512 | 35 | 2 | 60.94 | pittsburg-b.-T-OR-D | 102 | 7 | 2 | 85.29 |
| dermatology | 366 | 34 | 6 | 30.60 | pittsburg-b.-TYPE | 105 | 7 | 6 | 41.90 |
| echocardiogram | 131 | 10 | 2 | 67.18 | planning | 182 | 12 | 2 | 71.43 |
| ecoli | 336 | 7 | 8 | 42.56 | post-operative | 90 | 8 | 3 | 71.11 |
| energy-y1 | 768 | 8 | 3 | 46.88 | primary-tumor | 330 | 17 | 15 | 25.45 |
| energy-y2 | 768 | 8 | 3 | 49.87 | seeds | 210 | 7 | 3 | 33.33 |
| fertility | 100 | 9 | 2 | 88.00 | soybean | 683 | 35 | 18 | 13.47 |
| flags | 194 | 28 | 8 | 30.93 | spect | 267 | 44 | 2 | 79.40 |
| glass | 214 | 9 | 6 | 35.51 | spectf | 265 | 22 | 2 | 58.49 |
| haberman-survival | 306 | 3 | 2 | 73.53 | statlog-a. | 690 | 14 | 2 | 67.83 |
| hayes-roth | 160 | 3 | 3 | 40.63 | statlog-heart | 270 | 13 | 2 | 55.56 |
| heart-cleveland | 303 | 13 | 5 | 54.13 | statlog-vehicle | 846 | 18 | 4 | 25.77 |
| heart-hungarian | 294 | 13 | 2 | 63.95 | synthetic-control | 600 | 60 | 6 | 16.67 |
| heart-switzerland | 123 | 13 | 5 | 39.02 | teaching | 151 | 5 | 3 | 34.44 |
| heart-va | 200 | 13 | 5 | 28.00 | tic-tac-toe | 958 | 9 | 2 | 65.34 |
| hepatitis | 155 | 19 | 2 | 79.35 | trains | 10 | 32 | 2 | 50.00 |
| horse-colic | 368 | 25 | 2 | 63.04 | vertebral-c.-2clases | 310 | 6 | 2 | 67.74 |
| ilpd-indian-liver | 583 | 10 | 2 | 71.36 | vertebral-c.-3clases | 310 | 6 | 3 | 48.39 |
| ionosphere | 351 | 34 | 2 | 64.10 | wine | 178 | 13 | 3 | 39.89 |
| iris | 150 | 4 | 3 | 33.33 | zoo | 101 | 16 | 7 | 40.59 |
| lenses | 24 | 4 | 3 | 62.50 |  |  |  |  |  |

Table B2. Friedman ranking and average accuracy (%) for each classifier, ordered by increasing Friedman ranking.

| **Classifier** | **Rank** | **Acc.** | **Classifier** | **Rank** | **Acc.** |
| --- | --- | --- | --- | --- | --- |
| **MSNN (proposed)** | **30.00** | **83.40** | mda_caret | 62.48 | 72.39 |
| M_hopfield | 32.24 | 82.64 | LibSVM_weka | 63.29 | 77.11 |
| svmPoly_caret | 33.39 | 79.79 | RRFglobal_caret | 63.45 | 77.26 |
| rf_caret | 38.43 | 80.28 | SimpleLogistic_weka | 63.92 | 76.81 |
| avNNet_caret | 38.73 | 79.00 | multinom_caret | 63.93 | 76.61 |
| svmRadialCost_caret | 38.85 | 79.82 | rbfDDA_caret | 65.09 | 77.99 |
| elm_kernel_matlab | 39.83 | 80.52 | RandomForest_weka | 65.92 | 77.76 |
| pcaNNet_caret | 39.88 | 79.64 | gaussprRadial_R | 65.93 | 72.71 |
| svm_C | 40.16 | 79.91 | fda_R | 66.41 | 76.67 |
| parRF_caret | 40.25 | 79.57 | SelfNorm | 66.67 | 77.19 |
| svmRadial_caret | 40.41 | 79.50 | Bagging_RandomForest_weka | 66.96 | 77.33 |
| nnet_caret | 44.56 | 79.25 | lda_R | 67.23 | 76.47 |
| rforest_R | 45.99 | 80.05 | MultiBoostAB_RandomForest_weka | 67.41 | 77.51 |
| C5.0_caret | 47.25 | 78.15 | knn_R | 67.73 | 77.42 |
| mlp_caret | 49.76 | 78.52 | ldaBag_R | 67.89 | 76.60 |
| mlpWeightDecay_caret | 49.80 | 77.72 | svmBag_R | 68.59 | 75.26 |
| Bagging_LibSVM_weka | 53.07 | 78.53 | pls_caret | 68.65 | 73.08 |
| svmLinear_caret | 56.19 | 76.81 | Decorate_weka | 68.95 | 75.01 |
| RotationForest_weka | 57.87 | 77.89 | lvq_caret | 68.96 | 77.02 |
| pnn_matlab | 58.05 | 78.32 | lda2_caret | 70.67 | 76.16 |
| glmnet_R | 58.43 | 77.63 | gcvEarth_caret | 70.79 | 75.88 |
| cforest_caret | 58.84 | 76.87 | ClassificationViaRegression_weka | 70.88 | 76.87 |
| rda_R | 59.25 | 77.89 | LibLINEAR_weka | 70.89 | 76.35 |
| pda_caret | 59.95 | 77.33 | svmlight_C | 72.61 | 71.65 |
| knn_caret | 60.03 | 77.26 | MultiBoostAB_MultilayerPerceptron_weka | 72.71 | 76.83 |
| fda_caret | 60.37 | 77.42 | sda_caret | 72.83 | 76.23 |
| dkp_C | 60.47 | 78.41 | SMO_weka | 73.73 | 76.25 |
| MultiBoostAB_LibSVM_weka | 60.60 | 78.20 | mlm_R | 73.81 | 75.81 |
| RRF_caret | 60.73 | 78.78 | RandomCommittee_weka | 74.92 | 77.67 |
| rbf_caret | 61.61 | 76.42 | MS | 75.53 | 75.65 |
| adaboost_R | 62.15 | 76.93 | MultiBoostAB_PART_weka | 75.77 | 75.79 |
| mlp_C | 76.27 | 75.46 | nnetBag_R | 93.40 | 67.48 |
| hdda_R | 76.40 | 75.94 | Dagging_weka | 93.47 | 72.85 |
| elm_matlab | 76.79 | 76.46 | KStar_weka | 93.61 | 75.51 |
| MultilayerPerceptron_weka | 77.05 | 76.33 | PenalizedLDA_R | 94.49 | 71.59 |
| LN | 78.39 | 76.29 | MultiBoostAB_RandomTree_weka | 94.52 | 75.49 |
| MultiBoostAB_J48_weka | 78.48 | 75.69 | J48_caret | 94.63 | 74.71 |
| RandomSubSpace_weka | 78.75 | 75.73 | FilteredClassifier_weka | 94.73 | 73.56 |
| LogitBoost_weka | 79.11 | 76.17 | pam_caret | 95.13 | 73.12 |
| Bagging_PART_weka | 79.28 | 75.16 | rpart2_caret | 95.85 | 75.35 |
| HW | 79.67 | 74.17 | ctree2_caret | 95.92 | 72.71 |
| MultiClassClassifier_weka | 80.20 | 75.24 | qda_caret | 96.88 | 72.84 |
| mda_R | 80.45 | 75.74 | rpart_R | 96.96 | 74.20 |
| Logistic_weka | 80.71 | 74.95 | PART_weka | 97.15 | 73.83 |
| treebag_caret | 81.69 | 76.78 | NNge_weka | 97.28 | 75.25 |
| Bagging_Logistic_weka | 82.11 | 75.18 | PART_caret | 97.43 | 74.12 |
| Bagging_J48_weka | 82.45 | 75.09 | slda_caret | 97.56 | 72.19 |
| ctreeBag_R | 82.51 | 74.66 | rpart_caret | 98.44 | 73.84 |
| Bagging_weka | 82.52 | 76.72 | IB1_weka | 98.57 | 74.98 |
| AdaBoostM1_J48_weka | 83.07 | 75.88 | bdk_R | 99.15 | 75.06 |
| RBFNetwork_weka | 83.88 | 75.37 | naiveBayes_R | 99.99 | 73.36 |
| plsBag_R | 84.23 | 72.05 | nbBag_R | 100.39 | 73.30 |
| lssvmRadial_caret | 84.31 | 74.33 | simpls_R | 100.71 | 66.70 |
| Bagging_RandomTree_weka | 84.51 | 76.22 | MultiBoostAB_NaiveBayes_weka | 101.32 | 72.65 |
| MultiBoostAB_REPTree_weka | 85.28 | 75.67 | Ridor_weka | 102.04 | 74.04 |
| ResNet | 85.51 | 74.63 | NaiveBayes_weka | 102.15 | 72.71 |
| Bagging_DecisionTable_weka | 85.63 | 74.73 | kernelpls_R | 102.19 | 65.16 |
| BN | 85.72 | 73.04 | JRip_weka | 102.37 | 72.91 |
| Bagging_IBk_weka | 85.81 | 76.10 | IBk_weka | 102.49 | 74.11 |
| Bagging_LWL_weka | 85.81 | 76.10 | obliqueTree_R | 103.36 | 70.09 |
| MultiBoostAB_Logistic_weka | 86.08 | 74.61 | OrdinalClassClassifier_weka | 103.53 | 72.91 |
| END_weka | 86.71 | 75.77 | widekernelpls_R | 103.71 | 66.27 |
| lvq_R | 86.81 | 72.82 | REPTree_weka | 103.99 | 73.58 |
| AttributeSelectedClassifier_weka | 88.16 | 75.05 | stepLDA_caret | 105.39 | 71.79 |
| BayesNet_weka | 88.24 | 73.93 | rbf_matlab | 105.68 | 70.07 |
| JRip_caret | 88.65 | 73.55 | mlp_matlab | 106.27 | 70.97 |
| DTNB_weka | 89.20 | 74.59 | bayesglm_caret | 106.41 | 62.91 |
| MultiBoostAB_DecisionTable_weka | 89.36 | 73.52 | Bagging_NaiveBayes_weka | 106.68 | 71.25 |
| NBTree_weka | 90.52 | 74.52 | DecisionTable_weka | 106.95 | 71.87 |
| J48_weka | 90.67 | 75.00 | cascor_C | 107.24 | 73.09 |
| WN | 90.96 | 72.87 | mars_R | 107.49 | 66.11 |
| bagging_R | 91.00 | 67.92 | sddaLDA_R | 108.76 | 70.02 |
| C5.0Tree_caret | 91.07 | 73.84 | logitboost_R | 109.21 | 70.67 |
| C5.0Rules_caret | 91.99 | 74.80 | sparseLDA_R | 109.27 | 69.19 |
| ctree_caret | 93.17 | 72.79 | NaiveBayesUpdateable_weka | 109.51 | 70.02 |
| stepQDA_caret | 109.95 | 68.94 | ConjunctiveRule_weka | 131.85 | 63.31 |
| rrlda_R | 110.60 | 70.53 | VFI_weka | 137.07 | 66.77 |
| sddaQDA_R | 113.07 | 68.34 | Bagging_HyperPipes_weka | 137.59 | 62.03 |
| AdaBoostM1_weka | 113.33 | 66.63 | Bagging_MultilayerPerceptron_weka | 139.28 | 56.88 |
| glmStepAIC_caret | 114.48 | 61.87 | spls_R | 140.40 | 56.43 |
| LWL_weka | 116.47 | 69.72 | HyperPipes_weka | 143.00 | 60.30 |
| gpls_R | 116.65 | 58.50 | NaiveBayesSimple_weka | 146.77 | 46.05 |
| MultiBoostAB_weka | 120.11 | 65.86 | ZeroR_weka | 147.45 | 51.15 |
| glm_R | 120.97 | 53.83 | RacedIncrementalLogitBoost_weka | 147.45 | 51.15 |
| QdaCov_caret | 121.96 | 68.88 | MultiScheme_weka | 147.45 | 51.15 |
| MultiBoostAB_IBk_weka | 122.08 | 65.47 | CostSensitiveClassifier_weka | 147.45 | 51.15 |
| dpp_C | 124.45 | 66.33 | Vote_weka | 147.45 | 51.15 |
| RandomTree_weka | 124.64 | 72.19 | Stacking_weka | 147.61 | 50.62 |
| Bagging_OneR_weka | 126.76 | 67.21 | Grading_weka | 147.61 | 50.62 |
| Bagging_DecisionStump_weka | 128.09 | 64.95 | CVParameterSelection_weka | 147.61 | 50.62 |
| OneR_caret | 129.59 | 66.38 | vbmpRadial_caret | 147.63 | 32.96 |
| OneR_weka | 130.16 | 66.36 | MetaCost_weka | 147.85 | 51.13 |
| MultiBoostAB_OneR_weka | 130.89 | 66.67 | StackingC_weka | 148.03 | 50.56 |
| DecisionStump_weka | 131.21 | 63.50 | ClassificationViaClustering_weka | 161.92 | 54.27 |
