# Supplementary material for: Modern synergetic neural network for imbalanced small data classification
Source: Sci Rep. 2023 Sep 21;13:15669. doi: 10.1038/s41598-023-42689-8 (PMC10514188; doi:10.1038/s41598-023-42689-8)
Supplement: Supplementary file 2 — Supplementary Information 2. [file 41598_2023_42689_MOESM2_ESM.pdf]

Dataset	SNN	Mhopfield	SelfNorm	MS	HW	ResNet	BN	WN	LN	elm_kernel_matlab	AdaBoostM1_J48_weka	Bagging_DecisionStump_weka	Bagging_DecisionTable_weka	Bagging_HyperPipes_weka	Bagging_IBk_weka	Bagging_J48_weka	Bagging_LibSVM_weka	Bagging_Logistic_weka	Bagging_LWL_weka	Bagging_MultilayerPerceptron_weka	Bagging_NaiveBayes_weka	Bagging_OneR_weka	Bagging_PART_weka	Bagging_RandomForest_weka	Bagging_RandomTree_weka	DecisionStump_weka	MultiBoostAB_DecisionTable_weka	MultiBoostAB_IBk_weka	MultiBoostAB_J48_weka	MultiBoostAB_LibSVM_weka	MultiBoostAB_Logistic_weka
acute-inflammation	100	100	100	100	100	100	100	100	90	100	100	82.5	100	51.6	98.3	100	100	100	98.3	100	100	84.1	100	100	100	82.5	100	100	100	100	100
acute-nephritis	100	100	100	100	100	100	100	100	100	100	100	91.6	100	85.8	99.1	100	98.3	100	99.1	100	94.1	91.6	100	100	100	91.6	100	100	100	98.3	100
annealing	85.96882	90.87	76	73	36	26	12	65	50	97	87	83	77	76	95	87	95	91	95	48	84	83	82	95	94	83	88	83	87	95	81
arrhythmia	74.77876	73.45	65.49	63.72	62.83	64.6	59.29	60.18	57.52	71.5	71	55	65.4	62.1	72.7	74.7	70.5	63.7	72.7	54.2	62.3	55.5	73.4	59.9	68.5	55.9	67	56.6	73.8	69.2	57.3
audiology-std	79.59184	80.61	80	68	72	80	64	72	80	76	0	44	80	0	92	0	68	68	92	48	36	44	0	80	88	44	84	44	0	80	64
balance-scale	99.03846	98.72	92.31	92.31	91.03	91.67	92.31	95.51	98.72	91.8	78.8	66.4	84.4	7.8	84.3	82.5	99	89.1	84.3	83	89.6	69.1	85.4	84.8	82	59.3	83.2	73.4	82.5	97.9	89.4
balloons	87.5	60.25	100	50	25	100	100	0	75	93.8	68.7	50	56.2	43.7	56.2	56.2	43.7	56.2	56.2	56.2	43.7	56.2	56.2	50	56.2	43.7	31.2	43.7	56.2	62.5	62.5
blood	80.2139	82.09	77.01	77.54	79.68	80.21	76.47	75.94	71.12	77.5	76	76.2	76	76.2	76.7	76.4	78.8	77.5	76.7	76.2	74.8	75.9	77.4	73.3	71.9	76.2	75.4	76.2	77.2	77.4	77.9
breast-cancer	92.60563	76.22	71.83	69.01	74.65	74.65	73.24	61.97	66.2	74.6	70.6	73	74.8	70.2	73	70.9	73	72.7	73	70.2	72.3	72	69.5	72	68.1	72	76.2	72.7	71.3	74.4	72.3
breast-cancer-wisc	80.80808	97.42	97.14	97.14	97.71	97.14	98.29	96.57	97.14	96.1	95.9	93.1	95.7	94.2	95.1	95.9	95.5	96.4	95.1	65.5	95.8	93.9	96.1	97.1	95.7	91.1	96.2	94.5	95.8	95.4	96.2
breast-cancer-wisc-diag	97.4212	96.12	97.89	97.18	97.89	95.07	97.89	97.18	96.48	96.1	96.1	91.5	94.9	93.3	95	95.2	97.7	95.2	95	62.7	93.3	90.6	95.6	95.9	95.4	89.8	95.6	93.4	97.1	97.5	94.7
breast-cancer-wisc-	76.92308	83.84	67.35	73.47	83.67	81.63	77.55	83.67	79.59	82.7	74.2	76.2	76.2	75.7	75.7	77.2	77.2	73.2	75.7	76.2	72.2	75.7	76.2	77.2	78.7	76.2	73.2	75.7	77.7	76.2	75.7
breast-tissue	69.81132	79.25	73.08	46.15	61.54	42.31	46.15	53.85	57.69	70.2	66	43.3	63.2	65	66.9	72.6	66	68.8	66.9	16.9	66	56.6	70.7	69.8	70.7	40.5	62.2	40.5	68.8	67.9	68.8
congressional-voting	66.82028	64.52	61.47	60.55	58.72	59.63	58.72	58.72	57.8	61.2	61.6	61.1	62	60.9	59.7	60.9	60.9	60	59.7	62.2	57.2	62.9	60.6	60.2	59.3	61.3	61.3	61.3	61.1	60	59.3
conn-bench-sonar-mines-rocks	83.65385	81.73	78.85	82.69	84.62	80.77	71.15	82.69	67.31	90.4	82.2	70.1	76.9	69.2	74	77.8	87	72.1	74	46.6	71.1	68.7	76.4	84.6	80.2	72.5	75	73.5	80.2	87	70.6
conn-bench-vowel-deterding	97.37374	94.95	99.57	99.35	97.84	99.35	96.1	95.24	99.35	100	91.1	31.1	79.8	58.2	87.4	90.4	99.7	81.1	87.4	11.6	76.8	38.5	88.9	100	95.6	17.3	62.7	17.3	87.8	100	80.9
credit-approval	87.53623	86.67	84.3	84.3	85.47	84.3	87.21	90.7	85.47	86.6	85.3	85.5	86.5	45.5	86.2	86.8	85.5	86.5	86.2	85	77.3	85.5	86.9	87.6	85	85.5	85.2	85.5	86.3	85.9	85.9
cylinder-bands	75.39063	75.78	72.66	76.56	79.69	77.34	75	75.78	75.78	73.6	76.9	67.1	71.4	53.9	73.2	74	75.1	71.8	73.2	62.5	67.9	66.4	75.3	78.1	75.3	67.1	67.3	68.7	74.6	77.5	72.2
dermatology	98.9071	98.91	92.31	91.21	97.8	92.31	93.41	94.51	94.51	96.7	96.7	50.2	95.9	96.4	96.4	97.5	98	98.6	96.4	56	96.9	51.3	96.7	97.5	96.7	49.7	95.9	49.7	96.9	97.8	97.5
echocardiogram	83.07692	84.62	81.82	84.85	60.61	84.85	84.85	78.79	81.82	75	82.4	85.4	85.4	79.3	85.4	83.9	85.4	83.2	85.4	67.1	80.9	85.4	84.7	84.7	84.7	85.4	86.2	85.4	87	80.9	83.2
ecoli	88.69048	85.12	89.29	83.33	86.9	82.14	82.14	84.52	85.71	89.6	80	64.5	80.3	78.2	78.2	82.1	86.3	84.5	78.2	54.1	85.4	64.5	82.4	85.7	82.1	64.5	80.6	64.5	82.7	85.1	83.9
energy-	83.85417	83.85	95.83	95.83	88.02	81.77	86.46	90.1	94.79	91.7	97.1	81.2	91	51	95	96.3	95.9	88.1	95	80.7	82	83.7	96.7	96	97	81.2	95.5	81.2	96.4	94.9	88
energy-	89.58333	90.36	90.63	89.58	90.1	87.5	87.5	89.06	88.02	90.8	92.5	73.4	89.7	53.2	90.3	90.4	91.2	89.3	90.3	83.2	83.3	88.6	91.6	93.4	92.7	73.1	91	73.6	92.1	90.8	88.9
fertility	90	92	92	88	88	84	68	68	88	84	83	88	88	86	88	88	88	85	88	88	87	88	85	88	87	88	88	88	83	87	83
flags	52.57732	48.45	45.83	45.83	43.75	37.5	41.67	41.67	35.42	51	65.4	35	67.5	45.3	64.4	63.9	51	40.7	64.4	34	48.4	56.7	64.4	60.8	65.4	35	61.3	35	62.3	48.4	37.6
glass	67.28972	61.68	73.58	60.38	64.15	64.15	58.49	67.92	69.81	69.3	70.5	46.7	64.9	55.1	68.6	69.6	67.2	60.2	68.6	34.1	47.6	58.8	70.5	76.1	74.2	44.8	60.2	44.8	74.2	64	60.2
haberman-	76.47059	77.78	73.68	72.37	64.47	68.42	73.68	75	68.42	72.7	69.9	73.8	73.5	72.2	73.8	73.2	74.5	74.8	73.8	73.5	74.8	70.9	76.1	69.9	67.3	73.2	73.2	74.5	70.9	75.8	75.4
hayes-roth	90	83.75	67.86	46.43	78.57	71.43	75	57.14	89.29	85.7	92.8	53.5	78.5	53.5	75	78.5	89.2	53.5	75	50	64.2	78.5	82.1	89.2	89.2	53.5	46.4	53.5	85.7	82.1	53.5
heart-cleveland	68.21192	66.23	61.84	60.53	63.16	56.58	57.89	56.58	57.89	58.6	53.7	54.4	57.4	55.7	57	55.7	58.4	57.4	57	49.5	58	55.7	54.7	57.4	55.7	53.4	56.7	53.4	54.7	56.4	56.4
heart-hungarian	85.71429	85.03	79.45	83.56	79.45	80.82	84.93	75.34	84.93	82.5	77.5	78.9	78.5	69	80.9	79.5	81.9	81.6	80.9	68.3	82.9	78.9	80.9	81.2	78.9	78.9	81.9	80.2	81.6	81.6	80.6
heart-switzerland	45.90164	50.82	35.48	38.71	58.06	32.26	38.71	25.81	51.61	42.7	29.2	34.9	36.5	37.3	35.7	39	36.5	40.6	35.7	39.8	39	34.9	36.5	39.8	37.3	35.7	39	35.7	32.5	33.3	39
heart-va	36	36	36	26	40	26	28	22	24	31.5	35.5	27	30.5	25	35	38	31	32.5	35	33	32.5	30.5	33.5	35	32.5	28	28	28	34.5	34.5	31.5
hepatitis	87.01299	89.61	76.92	76.92	66.67	76.92	87.18	84.62	74.36	84	79.3	80.6	80.6	78.7	82.5	79.3	82.5	81.9	82.5	79.3	81.2	78	76.1	85.8	81.2	72.9	81.2	78	80.6	81.9	83.8
horse-colic	84.78261	84.24	80.88	85.29	77.94	80.88	85.29	70.59	79.41	88.2	72	77.9	85.2	64.7	85.2	70.5	89.7	88.2	85.2	66.1	73.5	76.4	72	86.7	82.3	77.9	85.2	82.3	83.8	89.7	88.2
ilpd-indian-liver	74.91409	75.95	69.86	66.44	67.81	67.12	59.59	69.18	69.86	72.8	67.9	71.3	72.5	72.2	69.2	69.6	69.2	72	69.2	29.5	56.4	69.6	69.9	70.4	69.9	71.3	70.6	71.3	70.4	68.6	72.5
ionosphere	98.85714	88.57	88.64	90.91	94.32	95.45	94.32	93.18	94.32	94	91.7	82.9	88.3	35.8	90.3	89.4	94.3	87.7	90.3	64.1	82	84	91.7	92	91.7	80	90.5	84.9	90.8	94.3	84.9
iris	100	100	97.3	91.89	83.78	97.3	91.89	100	97.3	98.6	94.6	72.6	95.3	92.6	95.3	95.3	94	94	95.3	33.3	95.3	94	95.3	94.6	94.6	66.6	95.3	94	94	94	94.6
lenses	91.66667	91.67	66.67	100	100	66.67	83.33	83.33	66.67	91.7	87.5	66.6	75	66.6	66.6	83.3	79.1	79.1	66.6	91.6	54.1	62.5	87.5	70.8	70.8	66.6	79.1	70.8	79.1	79.1	79.1
libras	83.33333	77.78	78.89	86.67	82.22	71.11	74.44	80	83.33	84.7	75.8	21.1	55	55.2	65.5	71.9	84.4	70.2	65.5	7.2	62.5	23.3	74.4	81.1	72.2	12.7	39.1	12.7	73.8	87.2	66.9
low-res-spect	95.09434	94.34	85.71	84.96	90.23	86.47	85.71	88.72	89.47	90.2	87.7	63.2	82.4	79.6	86	87	91.5	79.2	86	51.9	79.8	74.7	86.8	88.1	89.6	62.9	86.2	62.7	88.3	90.2	79.4
lung-cancer	75	81.25	62.5	37.5	12.5	25	50	50	25	37.5	46.8	62.5	50	62.5	62.5	59.3	43.7	50	62.5	46.8	59.3	46.8	56.2	40.6	50	53.1	56.2	53.1	59.3	37.5	40.6
lymphography	81.08108	85.14	91.89	72.97	72.97	67.57	75.68	75.68	78.38	87.2	84.4	66.8	76.3	66.2	79.7	77.7	83.1	81.7	79.7	74.3	83.7	77	81.7	85.1	82.4	68.2	78.3	66.2	81.7	87.8	79.7
mammographic	82.70833	84.17	82.5	80.83	79.17	78.33	81.67	82.92	82.08	81	79.2	81.8	83.4	56.9	82.1	82.9	83.3	82.3	82.1	59.2	78.7	81.8	82.8	79.6	78.6	81.8	82.8	81.8	81.3	83.5	82.5
molec-biol-promoter	90.56604	84.91	84.62	76.92	69.23	76.92	76.92	69.23	46.15	86.5	83	77.3	92.4	79.2	81.1	83	78.3	73.5	81.1	50	86.7	85.8	85.8	88.6	79.2	76.4	91.5	83	85.8	79.2	79.2
monks-1	100	100	75.23	65.51	58.33	75.46	90.74	50	70.14	66	72.2	58.3	58.3	41.6	61.1	72.2	52.5	58.7	61.1	50	59	58.3	72.2	61.1	61.1	58.3	58.3	58.3	72.2	56.9	58.7
monks-2	98	94.33	59.26	63.43	63.89	62.73	32.87	66.44	51.62	62.3	60.8	67.1	67.1	67.1	66.2	60.4	65.2	67.1	66.2	67.1	67.1	67.1	61.8	66.2	66.2	67.1	67.1	67.1	61.8	65	67.1
monks-3	99.27798	98.19	60.42	74.54	58.8	58.33	52.78	52.31	69.91	62.5	58.3	52.7	52.7	47.2	52.7	52.7	56.4	55.5	52.7	52.7	60.1	52.7	54.3	53.7	52.7	52.7	52.7	52.7	55.3	60.1	53.4
musk-1	92.43697	94.96	87.39	86.55	89.92	87.39	82.35	89.92	89.92	87.8	89.7	71.6	83.6	74.5	82.1	86.7	90.1	84.4	82.1	43.4	76.2	69.5	88.4	88	89.7	71.6	81.3	72.4	86.3	88.8	84
oocytes_trisopterus_nucleus_2f	85.30702	85.09	79.82	87.28	82.89	77.19	74.56	79.39	82.02	86.8	79.1	57.7	72.4	61.8	79	77.9	85.1	80.1	79	42.2	52.1	59.3	79.3	79.3	79.8	57.7	68.9	57.7	78	85.4	80
oocytes_trisopterus_states_5b	94.73684	93.2	93.42	94.3	93.42	89.47	89.47	92.54	89.91	94.4	90.6	78.8	88.8	78.1	89.1	90.5	94.1	91.4	89.1	76.7	76.7	82	91.9	90.4	90.6	78.5	88.5	79.1	91.6	93.8	91.7
parkinsons	84.53608	87.63	89.8	91.84	83.67	91.84	85.71	81.63	85.71	94.4	91.7	82	85.1	76.9	83.5	89.2	93.3	85.6	83.5	75.3	70.7	86.6	92.8	88.7	90.7	83.5	85.1	83.5	90.2	93.8	85.1
pima	74.21875	77.08	75.52	76.56	71.88	71.35	71.88	69.79	69.27	78.1	72.3	72	75.6	66.9	75	76.3	76.6	76.1	75	65.1	75.9	70.9	75.5	76.1	73.8	71.7	74.8	72.7	73.8	75.6	77.2
pittsburg-bridges-MATERIAL	90.56604	90.57	88.46	84.62	92.31	92.31	88.46	80.77	92.31	88.5	73.5	74.5	74.5	33.9	74.5	72.6	84.9	71.6	74.5	74.5	75.4	74.5	72.6	61.3	59.4	74.5	74.5	74.5	71.6	82	70.7
pittsburg-bridges-REL-L	72.54902	66.67	69.23	76.92	69.23	84.62	76.92	65.38	73.08	65.4	47.5	51.4	51.4	43.6	56.3	49.5	66	48.5	56.3	51.4	50.4	53.3	48.5	56.3	54.3	51.4	51.4	51.4	47.5	66	53.3
pittsburg-bridges-SPAN	78.26087	76.09	69.57	52.17	56.52	56.52	56.52	65.22	60.87	71.7	60.8	61.9	61.9	36.9	60.8	58.6	71.7	60.8	60.8	52.1	60.8	55.4	56.5	55.4	55.4	61.9	61.9	61.9	58.6	68.4	58.6
pittsburg-bridges-T-OR-D	88.23529	92.16	84	88	88	88	88	88	88	88	85.2	86.2	86.2	86.2	86.2	86.2	84.3	85.2	86.2	86.2	86.2	85.2	86.2	84.3	83.3	86.2	86.2	86.2	85.2	83.3	85.2
pittsburg-bridges-TYPE	55.76923	61.54	65.38	65.38	53.85	65.38	11.54	46.15	65.38	64.4	33.3	41.9	41.9	29.5	33.3	34.2	61.9	38	33.3	41.9	35.2	36.1	35.2	31.4	33.3	39	41.9	39	35.2	59	38
planning	75.82418	72.53	68.89	66.67	60	71.11	62.22	64.44	68.89	72.8	71.4	71.4	71.4	67	69.2	65.3	71.4	68.1	69.2	69.2	63.7	69.2	66.4	71.4	69.2	71.4	71.4	70.3	69.2	71.4	67
post-operative	80	73.33	72.73	72.73	59.09	72.73	59.09	54.55	77.27	72.7	55.5	71.1	71.1	70	70	67.7	70	58.8	70	64.4	63.3	70	58.8	65.5	57.7	71.1	71.1	70	61.1	63.3	57.7
primary-tumor	49.69697	43.03	52.44	50	45.12	39.02	51.22	50	45.12	52.7	45.1	27.8	45.4	25.4	46	43.9	49	38.4	46	36.6	46.3	28.4	43	43.6	41.2	27.8	42.4	27.8	42.7	45.4	38.1
seeds	97.14286	93.33	88.46	86.54	94.23	86.54	86.54	88.46	88.46	95.2	93.3	70.4	89.5	84.7	90.4	90.9	94.7	96.6	90.4	36.1	90	82.3	90.4	90	90.9	64.7	90	89	93.3	94.2	94.7
soybean	95.60117	95.6	85.11	87.23	86.17	86.7	88.83	85.37	84.84	90.4	87.5	26.3	76.8	13.8	83.5	80.8	91.7	86.1	83.5	53.1	87.2	31.6	70.7	90.1	89.6	26.5	85.1	26.5	86.1	89.8	87.2
spect	81.95489	75	63.98	61.83	60.22	66.67	63.44	63.98	67.2	58.3	67.9	55	55	59.8	55	65.7	55	58.8	55	55	55	55	68.4	54.5	58.2	55	55	55	63.1	55.6	58.8
spectf	75	81.95	49.73	60.43	89.3	70.05	22.99	45.45	55.61	91.4	91.9	90.9	91.9	91.9	91.9	91.9	91.9	88.7	91.9	91.4	91.9	91.9	91.9	91.9	91.9	90.9	80.7	91.9	91.9	91.9	88.2
statlog-australian-credit	67.82609	70.14	59.88	68.02	68.02	63.95	68.02	68.6	62.79	67.3	58.8	67.3	68.4	36	66.2	61.8	67.8	65.5	66.2	58.5	54.9	64.6	60.8	65.3	63.1	66.8	67.2	66.2	60.7	67.6	64
statlog-heart	85.18519	85.93	92.54	83.58	77.61	86.57	79.1	86.57	79.1	86.9	76.6	74.8	77.4	64	79.2	79.6	84.4	81.1	79.2	59.2	83.7	74.4	80.7	82.5	78.1	68.5	77.7	75.9	80	82.9	83.3
statlog-vehicle	75.1773	73.05	80.09	82.94	79.62	75.83	75.83	80.09	79.15	84.8	76.9	39.4	69.6	44.4	72.3	72.4	84	80	72.3	35.4	46.8	51.6	74.1	73.8	73.1	39.9	67.6	39.9	76.1	82.5	79.9
synthetic-control	97.66667	99	98.67	98	98.67	96	97.33	98.67	97.33	99.2	96.3	50.1	89.1	94.3	92	93.1	98.8	87.8	92	16.6	94.6	62	94.5	98.5	97	33.1	92.6	33.1	94.3	98.3	81.5
teaching	56	53.33	50	60.53	52.63	55.26	50	31.58	63.16	47.4	64.2	44.3	53.6	45	51.6	53.6	56.2	51.6	51.6	36.4	54.9	43.7	54.9	57.6	54.9	39.7	34.4	39.7	54.3	52.3	52.9
tic-tac-	100	99.16	96.65	98.33	97.49	96.23	98.33	97.07	97.91	99.9	98.6	69.9	79.8	34.6	97.2	97.5	99.4	98.3	97.2	65.3	70.4	69.9	98	97.5	96.6	69.9	75.3	67.9	97.5	98.9	98.3
trains	100	100	0	0	0	0	50	50	100	87.5	80	50	50	90	60	50	50	80	60	70	50	50	50	80	60	60	50	50	60	40	80
vertebral-column-2clases	86.45161	90.97	83.12	87.01	85.71	83.12	83.12	66.23	84.42	86.7	82.9	77	81.6	68.7	84.8	82.5	85.1	84.8	84.8	32.2	78	75.1	81.9	83.5	83.5	77.7	83.2	77	82.9	85.4	84.5
vertebral-column-3clases	84.51613	87.1	83.12	80.52	79.22	75.32	77.92	74.03	83.12	82.5	81.9	77.4	79.6	75.1	82.5	80.6	85.4	84.8	82.5	32.2	83.8	71.2	82.2	83.2	80.9	77.7	81.6	77.7	84.1	85.4	85.1
wine	96.62921	92.13	97.73	93.18	90.91	97.73	97.73	97.73	97.73	100	95.5	79.7	92.6	91.5	93.8	92.1	98.3	97.1	93.8	39.8	96.6	76.9	93.2	98.8	95.5	62.9	98.3	87.6	96	98.3	96.6
zoo	100	90	92	100	88	100	72	96	96	96	94	60.3	88.1	95	92	92	96	96	92	46.5	96	71.2	95	96	97	60.3	94	60.3	95	96	96

Dataset	MultiBoostAB_MultilayerPerceptron_weka	MultiBoostAB_NaiveBayes_weka	MultiBoostAB_OneR_weka	MultiBoostAB_PART_weka	MultiBoostAB_RandomForest_weka	MultiBoostAB_RandomTree_weka	MultiBoostAB_REPTree_weka	NaiveBayesSimple_weka	NBTree_weka	RandomForest_weka	RandomTree_weka	REPTree_weka	lda_R	lda2_caret	rrlda_R	sda_caret	slda_caret	stepLDA_caret	sddaLDA_R	PenalizedLDA_R	sparseLDA_R	qda_caret	QdaCov_caret	sddaQDA_R	stepQDA_caret
acute-inflammation	100	98.3	100	100	100	98.3	98.3	82.5	100	100	99.1	98.3	100	100	100	100	100	95.2	100	100	81.7	100	83.3	82.5	83.3
acute-nephritis	100	95	99.1	100	100	100	100	96.6	100	100	99.1	100	100	100	98.3	100	100	87.5	92.5	97.5	81.7	100	93.3	85.8	84.9
annealing	94	85	83	80	96	95	91	0	98	96	93	92	90	90	77	89	89	80	76	81	85	84	83	76	76
arrhythmia	69.4	62.8	55.3	75	60.8	64.8	70.5	0	57	60.3	55.7	68.8	50	40.3	63.3	67.2	67.5	57.1	58.4	70.1	56.9	58.8	6.4	58.8	0
audiology-std	68	80	44	0	76	88	84	0	72	80	72	84	56	56	56	44	44	64	8	52	60	52	52	8	0
balance-scale	91.3	90.4	74	86	84	80.8	85.7	90	78.5	84.3	79.3	78.4	87.7	88	60.6	88	64.2	87.2	70.2	88.1	69.1	91.4	92.3	87.7	91.7
balloons	68.7	56.2	50	56.2	62.5	68.7	62.5	0	56.2	62.5	62.5	50	75	56.2	68.8	72.1	50	56.2	75	87.5	50	62.5	75	68.8	74.2
blood	77.5	76	75.5	77.1	71.9	73.2	76.6	75.4	75	72.8	71.5	76	76.1	77	63.8	77	77.5	77	76.5	74.3	76.5	77.1	73.5	76.5	76.2
breast-cancer	68.5	72.7	69.5	72	70.6	68.1	72	72.3	72.3	73	64.3	72	70.5	71.3	70.8	72.7	72	70.6	70.1	71.5	68.4	69.2	71.2	69.8	71.3
breast-cancer-wisc	96.4	96.1	95.5	95.4	96.9	95.8	94.9	96.1	96.7	96.9	94.8	94.5	96	95.6	96.3	96	95.1	88.3	95	95.7	89.7	95.3	68.4	94.4	92.1
breast-cancer-wisc-diag	97.7	94.5	91.9	95.7	95.7	96.1	96.1	93.1	92.6	96.3	92.9	95.2	96	95.8	97.9	95.6	94.7	89.6	95.6	94.7	90	94.9	90.7	90.8	91
breast-cancer-wisc-	73.7	71.7	68.6	73.2	77.7	76.2	78.7	71.7	73.2	77.2	66.6	75.7	78.5	76.2	62	78.3	75.3	75.8	76	63.5	76.5	76.3	76.3	76	74.3
breast-tissue	65	66	54.7	68.8	71.6	68.8	71.6	64.1	66	70.7	66	70.7	73.1	69.9	74	62.4	53	62.2	69.2	60.6	71.2	64.9	74	67.3	61.4
congressional-voting	56.5	60.2	61.8	60	58.6	58.1	61.8	50.5	61.3	60.9	58.1	61.6	62.4	60.5	60.8	59.8	60.5	63	61.5	58.5	61.5	46.8	58.3	61.5	63
conn-bench-sonar-mines-rocks	83.6	74.5	68.7	81.2	84.1	77.4	74.5	69.2	77.8	84.1	64.4	64.4	71.2	71.6	76.9	77.4	78.4	73.5	73.6	68.3	69.7	69.7	61.5	75	70.7
conn-bench-vowel-deterding	98	80.9	32	90.9	99.7	94.3	86.7	75.9	87.6	99.7	83.3	72.5	73.4	74.5	72.3	73.4	35.5	57.4	47.8	40.5	17.3	98.3	76.6	35.1	81.8
credit-approval	85.9	78.4	85.5	85.7	87.6	86.9	86	80.5	85.7	88.1	80	84.6	87.1	85.9	71.5	85.9	82.3	85.5	85.9	87.9	85.9	81.4	85	85.9	85.5
cylinder-bands	74.6	69.5	66.4	78.1	78.3	75.9	71	65.6	71.6	78.9	67.9	70.1	73.6	73.2	67	72.1	65.4	66.4	63.1	68.6	68.4	73.8	62.5	61.1	66.8
dermatology	98	97.5	48.6	95.6	97.5	96.7	95	0	96.4	97.8	91.2	94.5	97.3	97.8	67.7	97	94.2	89.1	94	72.6	90.2	93.8	96.5	62	67.2
echocardiogram	78.6	79.3	80.9	85.4	83.2	84.7	83.9	85.4	83.9	84.7	75.5	85.4	85.6	84	73.5	81.7	78.7	85.5	85.6	85.6	85.6	85.6	53.8	72	69.5
ecoli	85.7	83	65.1	83.9	85.7	83.3	81.8	0	81.5	85.1	74.4	77.3	86.6	90.9	81.5	86.6	79.8	79.6	81.8	86.9	86.9	79.5	81	64	85.2
energy-	94.1	82.4	84.2	97.3	96.4	96.3	96	81.2	90.6	96.6	95.9	94.5	86.8	88.4	82.4	87	83.5	81.3	83.1	81.4	82.3	81.4	87.1	81.4	81.2
energy-	90.7	83.5	88.9	92.7	92.8	93.4	91.2	73.5	89.9	93	91.6	90.7	82.9	85.7	84.2	85.8	85.9	88.5	86.6	71.2	86.6	81.2	87.8	86.2	88.5
fertility	85	88	87	85	86	84	88	83	88	86	78	88	84	86	58	86	88	88	88	86	88	88	83	88	88
flags	47.9	45.8	57.7	67.5	60.8	62.8	60.8	0	59.7	61.8	50	57.7	45.3	42.7	33.9	48.4	50.5	41.4	32.3	46.9	45.8	41.1	40.6	34.9	31.4
glass	69.6	51.4	56.5	73.8	78.5	71.9	67.7	0	66.3	75.7	73.3	65.8	58.8	61.7	58.3	60.3	58.4	54.6	41.2	60.2	49.5	56.5	62	53.7	56.6
haberman-	72.5	75.1	73.8	74.1	70.5	67.9	73.8	74.8	71.8	69.6	64.3	72.8	74	74.5	68.8	75.1	73.5	73.9	70.7	74.7	74.3	75.1	70.1	73.7	73.5
hayes-roth	57.1	78.5	75	85.7	89.2	89.2	82.1	64.2	82.1	89.2	89.2	75	53.6	53.6	42.9	53.6	39.3	53.6	42.9	53.6	53.6	53.6	35.7	57.1	53.6
heart-cleveland	54.1	57	50.8	56.1	59	55.7	57	0	53.1	58	55.4	55.4	59.5	62.4	48.7	59.7	59.7	53.1	55.3	64.8	51.3	55.6	48	55.9	54.8
heart-hungarian	80.6	82.6	80.6	82.6	81.2	76.8	83.6	82.3	79.5	81.6	75.5	79.5	84.8	83	80.7	81.3	83.4	80.3	81.1	85.5	82.4	81.3	79.1	85.1	80
heart-switzerland	39.8	31.7	37.3	33.3	41.4	32.5	36.5	0	38.2	39.8	33.3	36.5	50.8	44.7	24.2	44.9	33.4	37.4	42.7	49.2	35.5	47.6	40.3	41.9	38.1
heart-va	30	33	28	35.5	38.5	29	30.5	0	29.5	38.5	26.5	30.5	31	38.5	29	31.5	36	32	25.5	31.5	30.5	25	32.5	31.5	27.5
hepatitis	84.5	83.8	82.5	81.9	84.5	81.9	80	36.7	80	87	72.9	81.2	80.1	79.3	77.6	79.4	81.3	81.3	79.5	83.3	78.8	79.5	78.8	79.5	83.2
horse-colic	80.8	70.5	72	79.4	85.2	73.5	86.7	75	77.9	82.3	76.4	83.8	89.7	89.7	75	85.3	85.3	82.4	82.4	85.3	82.4	80.9	83.8	83.8	82.4
ilpd-indian-liver	69.8	55.5	69.8	71.6	69.1	69.4	69.6	55.4	68.9	68.4	65.6	68.4	73.3	71.5	72.6	70.2	71	71.4	71.9	74.3	71.9	55.6	69.5	71.9	71.2
ionosphere	90.5	90.3	87.1	91.4	92.3	91.7	89.4	80	91.4	92.5	85.7	86.8	86.6	88.3	83.5	86.6	86.6	85.2	85.8	81.2	80.4	86.6	82.1	78.1	88.6
iris	95.3	95.3	94.6	94	95.3	94.6	95.3	95.3	94.6	94.6	94.6	96.6	99.3	98.6	97.4	98	91.3	96	96.7	97.4	97.4	98	58.3	94.1	93.3
lenses	83.3	62.5	66.6	83.3	79.1	75	79.1	0	58.3	79.1	66.6	79.1	87.5	79.5	83.3	82.5	70.4	66.8	66.7	79.2	91.7	83.3	83.3	66.7	87.5
libras	80.5	64.1	20.2	71.1	82.7	73.8	58.8	62.2	66.9	82.5	61.6	51.1	61.4	65.6	70.6	72.2	66.7	60	56.7	22.8	11.4	84.4	89.2	49.2	76.9
low-res-spect	91.9	81.7	73.8	88.3	88.3	87	85.6	0	82.6	88.7	84.3	82.6	83.8	84.8	86.3	85.1	85.5	83.3	79.9	78	86.3	75.9	69	55.6	88.7
lung-cancer	53.1	53.1	50	46.8	53.1	43.7	46.8	0	53.1	43.7	50	46.8	50	36.3	50	44.8	53.2	66.5	50	68.8	56.2	65.6	50	50	27.9
lymphography	81.7	85.1	77	79.7	87.1	81	72.2	0	76.3	86.4	75	71.6	86.5	81.7	70.9	85.2	85.8	75.1	56.8	82.4	73.6	75.7	77	56.8	80.7
mammographic	81.9	79.1	81.9	83.2	78.5	77.7	82.8	0	82.3	78.4	75.2	81.9	79.1	80	80.1	79.6	79.5	77.4	78.5	79.1	75.4	79.4	76.8	79	77.3
molec-biol-promoter	74.5	86.7	88.6	85.8	86.7	78.3	80.1	86.7	80.1	90.5	73.5	72.6	74	72.6	71.2	82.3	83	78.3	84.6	87.5	76	74	64.4	69.2	81.9
monks-1	70.3	59.7	58.3	72.2	61.1	61.1	61.1	56.7	61.1	61.1	57.4	50	59.5	59.5	64.8	58.6	58.8	61.1	63.9	59.7	66.7	56	65.5	66.7	58.3
monks-2	66.4	67.1	64.8	60.8	66.2	65.2	66.2	66.6	67.1	66.2	66.2	65.2	67.4	67.4	47.2	67.4	65	67.1	67.1	67.4	67.1	66.9	67.1	67.1	67.1
monks-3	54.8	66.6	52.7	56.9	53.7	53.7	52.7	52.7	52.7	53.7	62	52.7	56.9	56.9	52.8	54.2	57.4	52.8	52.8	56.2	63.9	69.4	70.4	52.8	52.8
musk-1	92.8	76.8	68.4	90.1	89.2	86.9	81	74.5	85	89.2	78.7	76.2	75.8	79	83	84.5	77.3	68.1	69.5	64.7	67	76.5	85.3	64.7	66.6
oocytes_trisopterus_nucleus_2f	82.6	53.3	58.6	78.8	79.9	75.4	79.7	52.3	74.8	80.5	71	71.7	79.7	79.6	75.4	72.3	62.2	66.9	57.7	58.7	55.2	72.8	70.9	59.6	59.6
oocytes_trisopterus_states_5b	94.6	76.4	81.3	92.6	90.6	89.2	88.5	76	86.5	90.6	85.7	88.1	93	92	92.2	92.2	78.5	78.9	80.8	74.1	82.5	0	0	83	79.9
parkinsons	90.7	69.2	84.6	90.7	88.7	91.2	86.1	68.7	90.7	88.7	86.6	84.1	88.8	87.7	80.1	83.6	83.1	86.2	74	76	85.7	88.7	81.5	83.7	84.7
pima	75.5	75.5	69.6	75.1	76.4	74	74.3	75.7	74.4	76.1	68.6	73.5	78.6	77.5	75.1	77.1	72.5	74.5	75.9	76.3	75.9	72.7	60.7	77.9	74.6
pittsburg-bridges-MATERIAL	72.6	74.5	74.5	74.5	62.2	62.2	74.5	0	74.5	63.2	54.7	74.5	82.7	84.8	66.3	85.8	86	86.9	81.7	85.6	81.7	81.7	83.7	75	84.9
pittsburg-bridges-REL-L	50.4	51.4	55.3	48.5	50.4	49.5	47.5	0	51.4	53.3	55.3	47.5	63.5	66	38.5	61.9	67.1	71	57.7	68.3	72.1	59.3	67.3	72.1	71
pittsburg-bridges-SPAN	55.4	61.9	48.9	61.9	53.2	58.6	59.7	0	60.8	61.9	52.1	59.7	71.7	71.5	57.6	69.3	63	65.2	58.7	65.2	67.4	65.3	57.6	66.3	55.4
pittsburg-bridges-T-OR-D	85.2	86.2	81.3	86.2	83.3	82.3	85.2	0	86.2	81.3	83.3	86.2	87.5	88.3	72.1	90.3	86.3	86.3	84.6	86.5	81.7	86.3	76.9	84.6	86.3
pittsburg-bridges-TYPE	36.1	33.3	33.3	41.9	32.3	31.4	38	0	39	36.1	32.3	38	62.5	61.1	27.9	56.2	57.1	58.9	50	60.6	64.4	51	58.7	52.9	54.5
planning	61.5	63.1	59.3	69.2	69.2	62.6	65.9	61.5	71.4	69.2	57.6	71.4	66.8	68.7	50.5	71.4	71.4	71.4	69.6	69.6	69.6	68.7	63.8	69.6	71.4
post-operative	55.5	58.8	62.2	54.4	58.8	60	71.1	0	71.1	66.6	58.8	71.1	63.6	70	47.7	66.7	71.1	68.2	72.7	65.9	67	61.4	56.8	72.7	71.1
primary-tumor	46	43.9	29.3	44.2	43	41.2	46.9	0	41.8	43	38.4	47.2	46.6	47.8	40.2	44.3	43	29.7	30.5	37.8	30.2	34.5	40.2	30.5	22.9
seeds	94.7	90.4	85.2	92.3	90	89.5	91.9	90	86.6	90	89	90.9	94.7	97.1	93.8	97.2	92.4	88.6	90.4	88.9	86.1	95.2	88.1	88.9	94.3
soybean	86.9	87.5	24.7	78.7	90.1	87.2	82.4	0	85.3	89.3	84.8	80.8	86.7	87	87.8	90.4	78.5	66.8	32.7	84.8	6.1	73.7	76.9	21	13
spect	62.5	55	55	65.7	55	51.3	55	0	55	54.5	59.3	55	61	61	63.1	58.3	59.4	57.8	57.8	61.5	57.8	45.5	51.9	55.1	55.1
spectf	90.9	91.9	91.9	91.9	91.9	91.9	90.9	0	91.9	91.9	87.7	90.9	85.6	85.6	92	92	92	92	91.4	92	46	78.1	81.8	92	92
statlog-australian-credit	60.2	53.1	64.6	61	64.6	64.4	68.4	52.3	67.9	65.2	56.8	67.3	66.4	66.1	53.6	66.2	67.7	67.8	68	66.9	68	52.6	55.5	68	67.8
statlog-heart	81.4	84.4	71.1	77.7	82.5	77.7	78.5	82.9	80.7	82.5	72.2	70.7	86.8	84.1	78.3	84.1	81.9	72.6	82.7	87.1	78.7	81.5	79.4	79	72.2
statlog-vehicle	82	45.9	53	75	73.6	74.2	72.5	46.9	69.9	74.8	68.4	70.5	78.2	78.7	76.5	79.1	43.7	54.8	58.8	33.5	58.1	84.5	77.7	47.8	59.2
synthetic-control	99.1	96.3	60.5	96	98.8	96.3	92.1	94.3	91.3	99	87.3	87	98.5	97.5	98.5	97.5	97.2	80.8	91.5	60.7	85	76.5	45.3	89	84.2
teaching	53.6	52.9	39	52.9	63.5	58.2	50.9	54.3	52.3	61.5	56.2	51.6	47.4	54.3	38.8	54.2	42.4	49.6	47.4	43.4	42.1	51.7	47.4	32.2	44.4
tic-tac-	98.4	74.2	69.6	98	97.9	96.2	97	70.3	69.9	98.4	89.8	93.4	97.6	98.3	96	93.3	69.3	69.9	64.3	71.4	68.1	98.3	97.6	69.1	69.9
trains	80	60	50	60	70	60	50	0	50	70	80	50	75	79.2	87.5	70.8	75	62.5	87.5	75	87.5	75	75	100	41.7
vertebral-column-2clases	84.1	79.6	75.4	81.9	82.9	84.5	83.2	78	82.9	83.2	78.3	77.7	82.7	85.2	84	83.5	70.3	76.1	74.7	67	70.8	82.9	72.9	77.2	78.4
vertebral-column-3clases	85.4	82.2	71.2	83.2	83.5	83.5	82.9	82.9	80	82.9	81.2	81.2	82.7	80.6	84.3	81	68.1	77.4	78.5	65.4	71.5	83.5	67.1	75.3	77.1
wine	96.6	96.6	81.4	96.6	98.8	89.3	91	97.7	97.1	98.8	93.2	89.3	99.4	99.4	98.3	100	95	91.6	96	87.5	88.6	98.9	89.3	95.5	91
zoo	96	96	71.2	95	97	95	94	0	95	96	95	90	99	96.1	96	93.2	93.1	84	88	82	93	93	91	74	90

fda_R	fda_caret	mda_R	mda_caret	pda_caret	rda_R	hdda_R	naiveBayes_R	vbmpRadial_caret	NaiveBayes_weka	NaiveBayesUpdateable_weka	BayesNet_weka	rbf_matlab	rbf_caret	RBFNetwork_weka	rbfDDA_caret	mlp_matlab	mlp_C	mlp_caret	avNNet_caret	mlpWeightDecay_caret	nnet_caret	pcaNNet_caret	MultilayerPerceptron_weka	pnn_matlab	elm_matlab	cascor_C	lvq_R	lvq_caret	bdk_R	dkp_C	svm_C	LibSVM_weka	LibLINEAR_weka	svmRadial_caret	svmRadialCost_caret	svmLinear_caret	svmPoly_caret	lssvmRadial_caret	SMO_weka	rpart_R	rpart_caret	rpart2_caret	obliqueTree_R	C5.0_caret	C5.0Tree_caret	ctree_caret	ctree2_caret	J48_weka	J48_caret	PART_weka	PART_caret	RandomSubSpace_weka	C5.0Rules_caret	JRip_caret	JRip_weka	OneR_weka
100	100	100	100	100	100	100	100	0	100	100	100	100	100	100	100	97.5	100	100	100	100	100	100	100	100	100	100	100	96	100	100	100	100	100	100	100	100	100	100	100	91.7	96.7	91.7	100	100	100	92.5	95.8	100	100	100	100	100	100	99.2	99.1	81.6
100	100	100	100	100	100	100	97.5	0	95	95	100	100	100	100	100	100	100	100	100	100	100	100	100	100	97.5	100	100	100	100	100	100	98.3	100	100	100	100	100	100	100	93.3	96	96.7	100	100	100	100	93.1	100	100	100	100	100	100	100	100	91.6
90	90	89	85	90	88	79	85	92	84	76	93	76	79	83	94	90	97	97	98	97	99	98	94	94	87	91	87	89	88	92	97	76	88	98	96	93	97	91	96	88	88	88	85	89	10	82	82	87	87	81	81	95	87	79	80	83
58.4	71.7	45.1	42.7	64.4	67	68.4	43.1	54.2	62.1	62.1	71.9	56.9	54.2	53.3	60.4	47.8	67.9	65	63.5	64.6	57.1	60.6	69.4	61.7	66.4	59.7	63.7	62.6	58.8	61.9	72.3	69.9	58.6	63.7	65.5	0	70.6	0	66.5	71.2	71.7	71.2	42.5	71.9	67	67.3	65.5	65.9	68.6	62.8	64.2	69	68.8	73.5	70.5	58.6
56	64	52	64	56	52	48	12	48	84	0	76	48	48	64	44	52	64	44	60	52	48	48	68	76	76	60	44	48	48	72	64	64	76	48	48	48	52	0	68	64	64	64	32	8	0	8	8	0	0	0	0	88	0	8	8	44
87.7	88.3	88.8	90.7	86.7	90.5	92.8	91.2	85	90	90	76	91.7	90.7	89.6	90.6	92.5	95.7	94.7	93.8	87.7	97.3	96.2	91.2	89.9	90.4	88.6	86.1	85.9	83.8	89.4	99	98.4	86.5	94.2	96.6	91.7	91.8	89.3	90.2	77.9	77.8	79.7	92.8	84	77.6	80.2	80.5	78.8	78.6	82.2	80.3	79.3	79.8	82.4	82.5	61.2
75	58.3	56.2	62.5	71.2	93.8	56.2	81.2	0	56.2	56.2	56.2	75	77.5	62.5	75	56.2	81.2	66.7	75	67.9	62.9	88.8	68.7	87.5	75	68.8	18.8	71.2	81.2	75	81.2	56.2	56.2	76.2	100	68.8	81.2	100	68.7	75	56.7	89.4	87.5	48.3	56.7	56.2	56.2	50	57.9	50	74.2	43.7	51.7	72.5	75	43.7
76.1	79.1	77	78.2	77	74.2	76.7	72.7	0	75	75	73.6	77.4	79.7	78.2	76.2	77.5	77.4	80.3	79.5	78.6	80.2	79.8	77	77.5	77.9	77	76.2	76.2	72.5	77.7	77.3	78.7	77.4	79.5	80.1	76.2	77.4	75.5	76.2	76.3	78.7	79.3	77	77.9	77	78.7	79.3	77.2	77	76.8	77	75.9	78.5	77.7	78.2	74.7
70.5	72.4	71.2	72.9	71.7	71.2	71.9	70.5	0	73.7	73.7	74.1	52.1	73.8	64.6	74.1	70.8	73.9	70.3	73.4	73.1	71.3	72	69.5	74.6	73.2	61.6	71.9	73.8	64.9	74.6	75	73.7	71.6	74.5	74.8	70.3	73.1	74.1	72	73.3	72.4	72.4	70.5	74.8	71.7	73.8	72.7	70.6	70.3	64.6	67.1	74.1	71.3	72.4	74.1	68.8
96	96.9	95.9	96.3	95.9	96.6	96.9	96.3	0	95.9	95.9	97.2	90.4	96.7	96.4	97	96.6	96.3	97	96.9	96.8	96.7	97.3	96.2	96.3	95.7	97.4	95.7	96.6	95.6	94.6	96.1	95.5	96.5	96.1	95.8	97	96.9	95.1	96.7	95.3	94.9	93.6	96.4	96.7	94.7	94.9	95.4	95.5	95.4	94.4	95	96.2	94.9	96	95.8	92.7
96	96	95.6	96	96.3	96.7	92.4	94.4	0	93.1	93.1	93.8	88.2	97.2	95.2	96.5	96	97.2	97.5	97.5	97.7	97.4	97.7	97.7	95.6	95.8	94.7	96	96.5	94.9	95.1	97	97.3	96.8	97.5	98.1	97.5	97.9	94.4	97.8	93.3	91	92.4	94.7	96	92.4	93.7	94.2	92.7	92.8	92.4	93.8	94.5	93.3	94.2	93.6	89.6
78.5	77.8	73	78.3	82.3	81	73.5	66.5	0	72.2	72.2	76.2	77.6	80.3	76.2	79.2	77	73	76.7	77.8	79.8	77.3	78.8	75.7	77.6	78.6	68.4	75.5	79.3	74.5	78.6	81.1	78.2	80.3	80.3	79.3	81.4	78.3	79.4	76.2	64	72.7	73.8	70	79.4	76.2	77.7	77.8	73.2	72.2	72.2	75.2	75.7	71.7	75.2	73.7	69.1
73.1	70	70.2	68	66.2	72.1	59.6	72.1	65	63.2	63.2	63.2	32.7	67	66.9	74.6	45.2	73.1	66.4	69.8	69.9	71.4	65.8	64.1	76.9	73.1	69.2	79.8	67.1	73.1	76	68.3	67.9	71.6	69.9	74.5	73.6	69.1	62.1	58.4	74	68.8	65.9	53.8	70	65.3	62.4	61.2	68.8	72.6	67.9	69.8	73.5	60.4	63.6	62.2	56.6
62.4	63	61.9	63.2	61.6	61.7	61.2	55.3	0	58.8	58.8	61.3	61.7	61.1	61.3	61.1	60.3	59.2	62.3	62.3	61.6	62.3	61.6	59.5	60.1	59.2	60.6	61.9	61.4	60.1	59.9	60.8	60.6	58.6	61.6	62.1	61.8	63	61.8	60.6	61.7	62.3	61.6	61.5	61.4	60.5	61.8	62.3	61.3	62.8	60.9	59.6	61.6	61.1	61.8	61.1	62.9
71.2	78.8	71.2	84.6	74.5	78.8	72.1	71.2	0	69.2	69.2	77.4	88.9	79.8	84.1	86.6	66.8	79.8	83.7	81.7	81.7	83.6	83.7	84.6	76.4	70.2	66.3	83.2	88	89.4	79.3	85.6	88.9	75.9	88.9	87	77	87.5	81.7	85	68.8	75	76.5	67.8	80.8	75.6	71.1	78.4	75.4	75.9	75	69.2	73	74	76.4	70.6	63.9
73.4	76.6	84.2	1.9	74.2	97	84.4	92.9	88.3	76.1	76.4	77	100	68.2	99.7	100	83.5	99.8	96.3	82	80.5	93.9	91.8	89.3	100	91.3	80.7	72.7	91.6	93.7	100	100	100	72.7	99.8	99.8	92.4	99.8	99.4	94.3	76.6	63.6	76.6	86.8	93.1	79.4	68.4	61.9	78.1	78.1	76.8	76.8	81.3	76.2	72.3	75.7	34.6
87.1	86.7	86.6	86.5	85.9	86.8	87.1	82	0	77.8	77.8	85.7	57	86.7	82.6	83.9	78.5	83.9	84.9	87	85.5	86.1	85.8	82.8	86.6	85.9	84.4	85	85.4	83.6	86.5	86.2	85.6	85.6	85.9	85.5	85.5	86.7	85.1	86.8	86.2	85.5	85.6	87.1	86.5	85.2	86.4	85.5	84.6	83.8	83.9	83.9	86.8	84.8	85.4	86.9	85.5
73.6	74.6	75.8	76.4	72.7	74.6	70.9	70.3	0	67.1	67.1	66	76.2	69.5	69.5	76.8	72.5	74.2	74.4	77.5	73.4	73.4	75	74.2	76.6	70.7	68	73.8	73.6	71.9	78.5	78.3	76.9	71.6	77.3	78.7	72.9	77.5	71.7	75.1	73.2	71.7	70.1	72.9	79.9	72.1	69.5	70.9	71.2	72.9	74.2	73	70.1	74.4	73	71.6	64
97.3	93.7	95.7	97.3	97.5	97.3	97.3	96.2	72.5	97.2	97.2	98.3	94.8	97	96.4	94	88.5	96.4	96.7	98.1	97.5	97	97	98	96.2	97.5	95.9	95.7	96.2	96.5	96.4	96.7	97.2	96.4	98.1	98.1	97.3	97.5	97	96.9	93.5	94.8	93.4	95.4	96.7	96.5	95.1	93.4	95.9	96.5	94.5	94.3	96.4	96.4	92.9	93.1	50.2
85.6	85.5	83.3	84	84.7	85.6	84.8	81.8	0	80.1	80.1	82.4	59.8	85.4	81.6	77.8	80.3	75.8	80.9	81	82.4	80.2	79.5	79.3	83.3	83.3	77.3	84.8	84	78.8	84.1	86.4	85.4	84.7	80.2	82.5	85.5	85.5	72.5	85.4	75.8	85.5	85.4	76.5	87.1	77	85.5	85.4	84.7	82.4	83.2	83.2	83.2	83.2	85.5	83.2	85.4
86.9	84.8	84.5	87.3	88.1	86.3	79.8	82.7	84.5	85.7	85.7	83	81.2	87.2	82.1	84.9	79.5	86	85.8	86.6	86	85.7	86	84.8	85.7	87.5	84.8	85.1	86.6	79.2	86.9	86.3	86.9	87.5	86.3	86.3	87.8	87.5	0	86	83	81.9	81	81.5	83.6	81.9	79.8	69.5	78.2	83.4	80.3	83.1	82.7	82.5	81.6	80	65.4
86.7	90.6	84.6	94.3	88.5	87.2	83.3	84.2	90.1	82.4	82.4	86.1	93.8	87.9	93.7	89.1	87.4	96.2	90.4	90.2	88.9	96.5	93.9	90.6	92.1	91.5	91.4	85.3	86.5	85.4	91.8	95.6	96.3	89	89.7	96.6	88.9	87.8	92.6	87.3	93.9	94.5	94.1	97	96.4	96.4	94.9	95.4	96.3	97	96.7	96.6	94.2	93.9	95.8	95	84.5
82.9	90.9	84.2	91	85.9	83.2	84.9	86.7	91.1	82.9	82.9	88.5	90.4	86.8	90.2	86.2	77.7	90.8	90.2	91.4	87.9	91.3	91.9	90.3	86.8	89.8	86.6	83.5	86.7	84.9	86.7	90.2	92.4	86.8	92.1	92.2	89.1	90.5	89.6	88.2	90.5	89.3	91.3	90.5	91.7	91.5	90.2	91	90.3	91	90.6	90.6	89.7	91.1	91.3	90.1	89
84	88	85	88	86	86	88	88	0	87	87	88	88	88	85	88	88	77	87	88	88	86	88	89	88	85	76	87	88	83	88	88	87	87	89	90	88	88	86	85	88	87	87	86	88	85	88	88	88	87	85	87	88	86	88	82	88
44.3	58.8	46.9	47.9	45.4	42.7	42.7	49.5	36.6	46.9	46.9	61.3	39.1	51.1	44.3	49.5	40.6	46.9	53.6	52.6	51.6	49	49.5	46.9	50	43.2	40.1	52.6	46.3	45.8	52.1	46.4	50	44.3	52.6	51	45.4	53.1	52.6	49.4	61.5	59.8	58.3	30.2	65	64.9	58.2	59.8	60.3	56.6	56.1	57.7	63.9	62.3	59.8	59.7	54.6
58.8	68.2	64.8	71.5	62.1	56.5	53.2	56	69.6	47.1	47.1	64	44.8	66.3	65.8	71.6	60.4	59	68.7	66.3	65.5	71.6	67.8	64.4	68.9	68.4	62.3	66.7	64.6	66.2	68.9	68.9	68.2	62.6	71.6	70.6	64.9	70.2	65.4	60.2	67.6	69.1	69.2	60.2	74.2	68.7	64.6	64	64.9	67.3	71.4	71.9	67.7	66.2	65.4	65.8	56
74	74.8	74.3	75.5	75.2	75	76	73.4	0	75.4	75.4	73.2	73.4	73.2	75.1	71.6	73.4	70.4	76.1	76.1	75.2	75.8	77.1	72.5	71.4	65.5	68.8	71.1	73.9	64.8	72.7	74	73.5	74.1	73.5	75.2	73.2	74.2	69.9	73.5	72.4	72.9	70.9	72	72.9	72.5	74.5	73.2	72.8	71.9	73.5	73.2	75.4	69	74.5	75.1	73.2
53.6	75	67.9	75	53.6	53.6	53.6	85.7	89.3	64.2	53.5	53.5	78.6	67.9	75	85.7	78.6	71.4	42.9	42.9	42.9	85.7	85.7	39.2	75	67.9	64.3	75	50	89.3	78.6	85.7	75	53.5	85.7	85.7	53.6	64.3	78.6	60.7	89.3	75	89.3	57.1	89.3	75	82.1	82.1	89.2	89.3	75	75	75	75	50	50	75
59.2	57.4	55.6	57.5	60.4	56.2	58.2	58.6	48.9	57	57	57.4	53	60.1	55.7	57.1	57.9	55.9	58.7	59.7	60.7	60.7	59	50.1	57.6	59.5	57.9	56.6	60.4	55.6	57.6	58.6	56.4	58.4	61.1	58.4	56.4	59.1	58.1	57.7	58.9	55.5	56.7	54.3	56.1	53.1	55.8	56.1	54.4	54.5	54.1	47.2	59	55.1	56.4	52.8	54.7
84.8	81	80.1	83.7	81	85.5	85.5	84.8	0	82.6	82.6	82.3	69.5	84	80.6	83.7	82.2	78.1	81.3	83.3	84.4	80.9	81.3	77.8	86	84.2	79.5	83.8	84.7	80.4	86.6	86	81.9	80.6	84.4	84.7	80.6	83.7	79.6	80.2	80.1	80.3	79.9	80.7	81.3	80.3	80.3	81.3	76.5	77.9	78.5	80.6	81.6	79.6	79.6	78.2	78.9
50	32.3	39.5	38.9	44	47.6	40.3	29	33.4	36.5	36.5	39	35.5	48	36.5	43.8	25.8	41.1	45.5	43.1	43.9	44.8	46.3	39	33.1	37.9	33.9	43.5	47.2	33.9	33.9	35.5	34.9	39	41.5	45.5	40.7	44.8	43.9	36.5	42.7	39	40.7	25.8	40	32.5	39.8	40.6	33.3	41.5	28.4	39	39.8	38.8	40.6	33.3	35.7
31	26.5	35	33.6	38.5	31.5	39	28.5	31	33	33	28	22.5	35.4	33.5	31.5	22.5	27	35	38.1	34	36	37.4	31	34	34	26.5	30.5	37.1	29.5	33.5	31.5	33	32	36.5	32	32.5	36.5	31.5	34.5	29	31.5	31.4	25	38	32	33.5	34.4	35	39.4	33.5	27.5	32	30	32	36.5	28.5
80.1	80	81.4	83.3	80	81.4	79.5	84	0	82.5	82.5	78	84	85.2	82.5	85.8	78.8	83.3	82.6	83.9	81.3	83.9	82	83.8	84	80.8	75	84	89.7	81.4	84	85.9	81.2	80.6	83.9	85.2	80.6	82.5	82	81.9	78.2	80	74.8	79.5	85.7	78.1	77.4	77.4	77.4	78.1	78	78	82.5	80.7	82.6	74.8	76.1
89.7	82.4	80.9	14.7	89.7	89.7	88.2	77.9	0	72	75	86.7	54.4	86.8	76.4	79.4	79.4	57.4	83.8	85.3	89.7	80.9	88.2	83.8	77.9	77.9	77.9	79.4	82.4	77.9	82.4	86.8	66.1	83.8	88.2	88.2	88.2	88.2	88.2	80.8	83.8	85.3	85.3	72.1	76.5	70.6	60.3	60.3	70.5	70.6	66.1	66.2	88.2	70.6	75	73.5	75
73.3	71.4	72.9	73.4	71.9	72.9	73.6	68.5	0	55.5	55.5	67.5	72.1	72.4	71.3	71.4	72.3	73.8	73.1	72.2	71.5	72.2	71.9	71.8	72.3	72.4	70.2	66.6	69.8	69.9	72.6	71.1	68.6	73.5	72.4	71.4	71.4	71.4	70.5	71.3	71.2	68.4	70.8	74.1	70.7	70.5	71.4	71.4	71.5	67.6	69.8	70	71.6	71	69.8	70.1	67.9
86.6	90.3	86.6	89.5	86.9	91.8	94.3	88.9	0	81.7	81.7	89.7	71.6	94	90.5	95.2	86.9	90.1	91.4	92.6	89.2	91.2	91.7	91.4	92.9	88.4	84.1	85.5	86.9	86.4	79.8	94.9	94	88.3	95.2	95.5	88	92.3	91.4	90.5	86.4	89.2	87.5	85.8	94.6	90.3	90.9	91.2	90.5	89.1	88.8	88.9	89.7	91.4	89.8	88.8	80
99.3	96	98.7	97.4	96.7	99.3	98.7	97.4	91.3	94.6	94.6	94.6	90.5	94.7	95.3	94.7	79.7	95.9	96.7	97.3	96	96.6	97.4	95.3	95.3	90.5	99.3	96.7	94.6	93.4	96.6	96.6	94	93.3	96	94	98	98	94.6	96	96.7	92.7	95.3	98.7	95.3	90.7	95.3	94.6	95.3	92.7	94.6	96.7	95.3	95.4	95.3	95.3	94.6
87.5	87.5	87.5	84.5	79.2	83.3	83.3	79.2	84.5	54.1	54.1	58.3	62.5	87.5	87.5	83.1	58.3	75	79.5	87.5	78.9	81.7	84.5	79.1	87.5	83.3	70.8	79.2	76	79.2	87.5	83.3	79.1	83.3	83.1	83.3	78.1	88.1	83.3	87.5	66.7	62.6	62.6	16.7	87.5	83.7	62.5	62.6	83.3	81.7	83.3	83.3	62.5	86.4	78.1	75	62.5
61.4	71.1	79.7	83.6	72.5	76.9	87.2	65	45.6	61.9	61.9	58	81.9	62.2	79.1	85.6	26.9	77.2	76.9	73.6	74.2	77.8	76.1	78	84.7	77.5	57.5	64.4	75	76.9	84.4	88.6	85.8	71.1	83.9	86.1	80.3	82.5	83.9	82.7	51.4	55.3	52.5	25.3	80.6	59.7	45.8	45.8	64.4	68.3	60.5	66.4	60	64.7	50.6	45.8	19.7
83.6	87	85.3	84.9	88.3	88.2	81.4	79.5	59	80.4	80.4	79.8	84.8	86.6	85.6	84.2	87.6	92.3	91.2	90.6	89.1	91	89.6	91.7	88.9	89.1	83.1	87.4	86.1	86.8	88.7	93.4	90.7	87	87.4	88.3	90.4	90.8	87.6	91.7	84	83.8	82.1	65	87	83.8	81.7	82.5	82.4	85.1	83.9	83.1	85.6	82.5	84.4	84.7	74.9
50	56.2	37.5	0	52.3	53.1	46.9	46.9	28.2	59.3	59.3	53.1	34.4	62.5	59.3	50	40.6	28.1	59.1	52.4	55.6	59.4	57.1	53.1	56.2	53.1	56.2	50	73.4	37.5	53.1	62.5	50	46.8	50	44.6	45.8	56.2	50	50	46.9	37.5	46.9	25	59	59.3	46.4	49.4	53.1	32.9	46.8	40.5	43.7	51.2	72	53.1	50
86.5	80.5	79.1	81.1	86.5	87.8	89.2	81.1	62.2	83.1	83.1	75.6	72.3	87.1	82.4	83.1	78.4	83.1	85.7	81.8	83.2	81.2	83.1	83.7	86.5	75.7	79.1	79.7	82.5	80.4	85.8	87.2	85.1	82.4	83.8	85.9	81	85.1	80.4	84.4	71.6	71	74.9	10.1	85.1	76.2	64.2	76.4	77	75.1	81.7	75.7	80.4	75.7	77	74.3	75.6
79.1	83.5	77.9	81.8	80.6	79.7	78.3	82.5	0	78.5	78.5	83.1	80.7	82.7	78.5	79.9	79.8	83.6	84	83.7	83.7	83.8	83.7	81.1	79.4	80.8	80.3	79.2	79.6	79.3	79.1	82.5	82.9	81.8	82	82.1	82.1	83.4	81.4	81.6	82.3	81.9	82.4	82	83.6	82.5	81.9	82.4	82.6	82.5	83	82.2	83.2	82.7	82.5	82.4	81.8
74	90.6	74	72.6	79.4	85.6	81.7	93.3	0	87.7	87.7	89.6	76	79.2	84.9	85.8	77.9	89.4	80.2	82.9	78.4	79.3	80.1	74.5	78.8	77.9	80.8	80.8	84.9	77.9	77.9	86.5	80.1	78.3	82.1	81.1	78.3	85.1	80.3	78.3	76	76.4	74.5	70.2	86.9	83	74.4	77.4	79.2	64.4	77.3	77.4	80.1	76.4	79.3	79.2	76.4
59.5	58.3	67.6	30.8	59.7	55.8	59.5	42.1	0	59.2	56.4	58.3	50.9	50	63.1	58.1	65.5	59	78.7	78.2	78.7	79.9	69.9	49.3	64.6	57.9	50.9	59.3	66.9	63.9	61.6	51.9	51.8	59.9	51.6	64.1	63.9	55.6	50.9	58.1	60.2	61.1	61.1	71.3	72.2	72.2	72.2	72.2	72.2	72.2	72.2	72.2	58.3	72.2	50	66.6	58.3
67.4	67.1	66.7	1.9	67.1	67.1	66.9	65	0	66.8	66.8	67.1	65	65.5	65	66.7	66.2	65.7	66.9	66.4	67.1	65.3	66.4	66.4	65.7	61.1	62	65.3	67.8	65	65.7	65.3	65.2	67.3	66	65.5	67.1	66.9	64.6	57.4	64.8	64.8	64.8	66.7	62.5	64.4	67.1	60	64.3	64.4	65.7	65.7	67.1	64.8	59.7	67.1	67.1
56.9	52.8	63.4	56.7	56.9	64.6	68.5	47.2	0	66.4	52.7	52.7	0	52.8	62	52.8	53	40.3	55.3	60.4	66	58.1	60	58.7	55.6	46.8	57.4	56.9	53.7	57.6	55.3	53.2	53.2	56.9	47.9	48.6	60	67.6	45.6	53.4	52.8	52.8	52.8	59	53.9	52.8	52.8	52.8	52.7	52.8	54.6	54.6	52.7	52.8	52.8	52.7	52.7
75.8	84.5	78.8	86.3	83.4	80	83.4	76.3	0	74.7	74.7	82.7	89.5	82.3	85.2	89.9	79.2	88	91.2	92.2	92.4	89.9	91.6	92.8	85.9	78.6	73.3	84.7	86.6	85.5	86.6	87.8	90.3	85.2	93.5	93.7	86.6	92	85.5	91.3	72.7	76.5	74	73.3	92.4	79.6	74	71.6	81.7	80.7	85	82.6	82.5	85.7	74.6	75.8	61.5
79.4	83.3	76.5	79.9	80	75.9	69.5	62.6	0	52.1	52.1	61	76.5	77.2	57.7	76.3	76.8	82.3	83.7	85.3	80	84.4	80.5	82.4	77.4	81	76.2	21.4	68.3	71.5	76.6	86	79.7	79.6	83.1	85.5	81.3	85.1	77.6	81.2	73.5	78.2	75.8	80.3	81	72.3	70.9	74.8	73.1	71.4	71.6	72.8	75.9	75	74.1	72.6	58.2
93.2	93.2	91.2	92.8	92.8	91	85.9	78.6	87	76	76	81.4	83.4	90.9	81.7	91.1	89.3	94.3	93.8	94.2	91.6	94.1	92.3	94.5	89.9	93.2	92.2	0	88.4	86.2	89.9	95.1	91.7	92.6	92.4	94.1	93.1	93.5	91.1	92.7	87	86.5	87.6	93.5	93	88.7	86	86.1	89.1	88.5	89.6	90.1	89.4	90.6	88.2	88.5	78.5
88.8	88.2	89.8	91.8	85.6	86.2	81.1	73	0	69.2	69.2	78.9	80.6	88.2	83.5	93.8	87.8	89.8	94.3	90.3	89.2	91.3	90.3	88.7	92.9	86.2	80.6	89.8	89.7	91.8	92.9	93.4	93.8	87.6	92.8	93.3	87.7	91.3	90.7	88.2	83.7	85.7	86.7	81.1	90.8	83.1	84.6	84.6	84.6	85.7	88.2	87.7	85.6	83.6	91.8	86.6	84.6
78.6	77.2	75.5	76.6	77.5	78.1	78.6	75	0	76.1	76.1	74.7	72.1	77.6	74.3	75	74	77.9	75.5	77.1	77.6	77	77	75.7	77.1	76.4	73.3	75.5	75.4	68.8	72.4	78.3	75.9	76.5	76.4	77.2	76.8	78	75.4	77.8	76	75.1	74.7	78	76.4	76.3	74.9	75.1	73.3	73.3	73.6	70.4	73.9	73.2	76.2	73.8	70.1
82.7	85.9	76	84.9	85.9	83.7	84.6	91.3	77.4	83.9	83.9	86.7	78.8	84.9	85.8	84	77.9	56.7	84.9	86.8	86.8	86.8	86.8	79.2	78.8	84.6	71.2	88.5	86.8	72.1	79.8	79.8	83.9	83.9	86.9	85.9	83.9	86.8	85.8	74.5	89.4	86.9	86.8	60.6	86.8	86.8	86.8	83.1	87.7	85.9	86.7	80.3	86.7	84.9	86.8	80.1	86.7
63.5	70.9	67.3	76.9	67.2	67.3	63.5	66.3	69.7	63.1	63.1	67.9	57.7	68.1	66.9	67.9	54.8	62.5	75.7	67	69	71.9	70.8	60.1	68.3	64.4	59.6	79.8	70.9	69.2	69.2	72.1	67.9	62.1	72.6	70	66	69.9	71.7	51.4	73.1	63.2	70.8	56.7	69.8	67	70.9	71	67.9	67	66	66	70.8	68.8	70.9	67.9	70.8
72.8	68.6	68.5	63	69.4	75	67.4	71.7	69.9	71.7	71.7	61.9	43.5	66.4	69.5	61.1	62	63	71.9	71.7	70.7	73.8	65.4	64.1	76.1	68.5	55.4	72.8	74	67.4	76.1	80.4	70.6	70.6	68.4	69.6	71.6	75	60.7	61.9	52.2	61.8	59.8	64.1	71.8	66.2	63.1	62.1	65.2	55.4	58.6	58.2	63	62.8	60.9	60.8	54.3
87.5	85.3	88.5	93.5	89.3	85.6	82.7	85.6	0	89.2	89.2	86.2	84	89.2	85.2	88.2	84	81	87.2	90.3	88.2	89.2	87.3	85.2	91	87	76	85.6	90.2	86.5	88	80	84.3	86.2	86.3	89.2	91.2	87.3	83.4	86.2	80.8	86.3	80.3	83.7	86.3	86.3	86.3	86.3	83.3	82.3	84.3	83.3	85.2	83.3	86.3	84.3	85.2
60.6	57.1	64.4	59.1	57.8	64.4	64.4	67.3	49.6	57.1	57.1	51.4	15.4	60.9	55.2	64.7	43.3	64.4	65.8	62.1	56.3	61.1	59.7	53.3	73.1	61.5	46.2	62.5	58.2	65.4	68.3	76	60	58	59.1	60.8	57.4	58.1	54.2	41.9	63.5	54.8	58.2	40.4	62.8	50.7	57.3	54.3	57.1	49.6	58	58	54.2	49.8	55.3	55.2	56.1
66.8	71.1	64.1	69.2	70.3	65.8	59.8	55.4	0	59.3	59.3	71.4	72.8	70.3	62.6	70.3	68.3	42.8	65.3	71.4	71.4	69.3	71.4	58.7	72.8	71.1	58.3	63.6	69.8	58.2	71.1	72.8	71.4	69.7	71.4	71.4	71.4	71.4	62.1	70.3	59.2	62.1	59.4	67.9	71.4	71.4	71.4	71.4	71.4	71.4	71.4	71.4	71.4	71.4	71.4	71.4	59.8
63.6	70.2	58	59	71.2	69.3	60.2	71.6	54.3	62.2	62.2	71.1	61.4	64.4	51.1	61.1	71.6	38.6	65.6	68.9	71.1	67.8	71.1	53.3	72.7	68.2	63.6	72.7	71.1	53.4	72.7	70.5	70	63.3	71.1	71.1	70.1	71.1	56.7	68.8	72.7	71.1	74.2	62.5	71.1	70	71.1	71.1	71.1	69	58.8	56.7	71.1	71.1	71.1	71.1	70
45.1	40	37.8	40.6	44.5	48.8	49.1	30.5	36.3	43.9	43.9	41.8	38.1	48.2	26.3	40.3	28.7	46.3	47.3	46.1	48.2	43.4	45.8	40.9	43.6	50.6	41.5	40.2	44	35.1	43.6	47.3	47.2	46.6	47.6	47.3	47.5	46.1	44.2	40.3	49.4	46.3	46.7	25.6	45.8	45.8	40.3	40	45.1	41.2	41.2	43.3	43	43	40	40	28.4
94.7	94.3	94.2	94.8	95.7	93.3	93.8	89.9	93.3	89.5	89.5	88.5	95.2	95.2	92.8	91.9	90.4	87	96.7	96.2	95.2	97.2	95.3	93.8	92.8	95.7	90.4	93.8	93.3	90.4	89.4	95.7	94.7	94.7	93.8	93.4	96.6	93.8	93.3	92.3	89.4	89.6	89.5	93.3	92.9	92.7	86.7	88.1	89	91.9	90.4	93.4	90	89	91.5	86.6	83.3
86.7	90.7	86.2	88.8	86.4	91.2	85.4	80.1	74.7	86.7	10.3	88	80.6	82.4	86.7	82.4	34.6	90.7	89.4	85.6	87.8	89.1	91	86.9	87.2	84.8	85.4	80.3	84.8	80.9	87.2	88.6	48.6	87.7	90.7	88	87.2	89.4	89.9	87.5	71.8	73.4	71.8	79.3	58.8	81.4	57.4	57.4	81.1	81.1	52.3	52.4	86.1	75.3	46.5	65.6	31.6
61	60.4	56.7	7.5	59.4	59.4	59.4	54	0	55	44.9	55	42.2	55.1	57.2	61	57.2	55.1	63.1	59.9	55.1	61.5	58.8	62	55.1	56.1	59.4	55.6	55.6	55.6	55.1	55.1	54.8	62	55.1	55.1	57.8	55.1	65.2	56.1	55.1	55.1	55.1	44.9	57.8	66.8	72.2	72.2	66.8	66.8	68.9	69	55	66.8	55.1	55	55
85.6	90.9	84.5	5.9	92	92	88.2	79.1	0	91.9	91.9	91.9	92	57.2	91.9	92	92	60.4	72.7	71.7	58.3	74.9	92	90.9	74.3	75.9	56.7	92	89.8	92	92	92	91.9	66.3	92	92	92	92	92	68.4	92	92.5	90.9	86.1	92	92	90.9	92.5	90.9	90.9	92.5	92.5	91.9	92	89.3	91.4	90.9
66.3	68.4	66.3	66.7	66.4	66.7	66.1	67.9	0	54.6	54.6	67.8	68	68	54.3	65.7	67.9	68	67.8	67.8	67.8	67.4	66.7	61.4	67.6	67.9	59.7	65.4	68.8	59.2	67.7	68.2	67.6	66.9	67.8	67.8	67.8	67.8	65.9	66.9	67.6	68.3	69.1	66	67.5	68	67.8	67.2	63.6	62.6	59.7	62.3	67.9	66.8	68.1	66.6	67.6
86.8	82.2	80.9	81.5	83.7	85.7	86.4	86.4	0	84.4	84.4	80.3	71.3	84.8	82.2	85.2	75.4	80.6	83.7	85.6	83.7	83.3	84.1	78.1	84.3	85.1	79.5	84.6	84.1	84.9	85.1	88.1	84.4	84.4	83.4	84.1	83.7	85.6	78.5	80.7	79.4	80.7	78.5	82.7	81.1	74.8	77.1	78.5	77.4	79.3	77.4	76	75.5	81.8	80	72.2	69.6
78.2	79.7	81.8	81.3	78.8	78.7	66	63.3	68.4	46	46	59.1	75.1	74.9	70.9	70.2	71.3	80	82.9	82.6	78.6	82.2	77.5	80.7	70.7	80.5	74.3	66.9	67.1	69.1	70.4	84.7	83.2	79.4	81	84.2	79.5	85.1	78.6	80.1	67.9	68.2	68.1	75.4	76.2	71.6	68.8	69.7	74.9	69.6	72.3	72.2	73.6	72.6	68.7	68.4	52.9
98.5	97	98.3	99.5	97.2	99.2	98.5	98.2	17.7	94.3	94.3	96.5	96.8	96.3	99.1	96.7	91.2	99	99	98.7	98.8	98.8	99.3	99.1	97	93.2	90	96.5	98.3	96.2	96.7	99.5	99.1	92.1	99.7	99.5	98.7	99	97.2	98.5	87.3	86.3	86.7	95.3	97.8	91.8	86.8	84.5	89.6	90.7	89.5	91.3	93.1	91.2	83.5	86.8	52.5
47.4	42.5	46.7	53.7	56.2	44.7	46.1	44.7	56.9	52.9	52.9	34.4	50	55.6	60.9	60.8	40.1	46.1	57.1	60.3	53.6	61	60.4	46.3	53.9	45.4	54.6	45.4	53.6	46.7	53.3	48	55.6	52.9	61.5	55	52.2	56.9	49.7	50.3	45.4	51	50.9	40.1	57.7	54.3	52.9	46.4	54.9	60.2	52.9	54.3	45	51.6	44.4	41.7	45
97.6	82.8	97.6	98.4	98.3	84.7	97.6	67	0	70.1	70.1	69.9	99.3	87.5	98.6	98.7	97.9	97	98.4	98.3	98.3	98.4	98	97.9	100	97.9	97.6	97	75.5	96.7	100	100	99.4	98.3	99.6	99.6	98.3	99	97.5	98.6	89.7	92.6	92.2	97.6	98.6	93	92.6	93.5	93.7	94.7	97.7	96.5	81.8	98.2	98.3	98	69.9
87.5	75	75	100	83.3	87.5	87.5	87.5	0	60	60	50	62.5	66.7	30	83.3	62.5	87.5	79.2	87.5	79.2	79.2	91.7	80	50	50	87.5	12.5	79.6	75	75	50	40	80	87.5	62.5	70.8	75	62.5	70	50	41.7	98	87.5	79.2	70.8	41.7	41.7	80	87.5	80	83.3	60	81.2	41.7	50	40
82.7	84.2	82.4	86.8	84.5	77.2	78.5	74	0	78	78	78	84.7	85.8	80.9	84.2	79.2	82.1	86.1	86.1	84.8	86.1	86.4	84.8	81.5	85.7	79.9	79.2	77.1	79.2	82.1	85.4	85.4	84.5	85.5	86.5	85.8	86.1	81.9	77.7	80.1	82.3	82.9	84.6	85.2	80.7	81	82.3	80.3	79.3	80.6	81.3	79.6	80	83.2	80.3	76.7
82.7	82.6	84	84.2	81.3	81.7	82.7	77.9	81.3	83.5	83.5	76.4	43.2	85.4	83.8	81	61	84.4	84.8	86.8	85.5	87.4	85.2	84.8	80.8	83.1	80.2	77.9	81	78.5	81.2	83.8	85.8	86.1	83.9	83.9	87.4	85.5	81.3	76.1	81.7	82.9	81.3	83.7	84.2	79.7	80.3	83.5	80.9	81.3	81.6	80.7	76.4	79.7	80.6	79	73.8
99.4	97.7	98.9	99.4	98.3	99.4	99.4	97.7	91	97.1	97.1	98.8	86.9	98.9	97.1	97.2	97.7	99.4	97.7	98.9	97.8	98.9	98.3	96.6	97.2	92.6	96	95.5	97.8	97.2	96.6	98.3	97.7	97.7	98.3	98.9	98.3	98.3	97.2	97.7	84.7	88.2	89.9	97.7	98.3	94.9	89.3	88.8	91.5	91.6	92.6	92.1	92.6	92.1	92.7	89.8	75.2
98	96	96	98.1	98	96	95	91	94.1	96	96	97	98	92.2	95	95.2	72	95	93.1	93.9	93	97	95.2	96	96	97	97	95	93.2	96	98	98	96	97	94.2	92.3	96.2	94.2	92	96	87	84.1	86.2	63	95.1	95	85.3	84.2	93	95.1	94	93.1	91	93.1	90.2	92	71.2

OneR_caret	Ridor_weka	DTNB_weka	ZeroR_weka	DecisionTable_weka	ConjunctiveRule_weka	adaboost_R	AdaBoostM1_weka	logitboost_R	LogitBoost_weka	RacedIncrementalLogitBoost_weka	MultiBoostAB_weka	bagging_R	Bagging_weka	treebag_caret	ldaBag_R	plsBag_R	nbBag_R	ctreeBag_R	svmBag_R	nnetBag_R	MetaCost_weka	Stacking_weka	StackingC_weka	rforest_R	rf_caret	RRF_caret
80.8	100	100	50.8	100	85	98.3	100	100	100	50.8	100	94.2	98.3	99.2	100	100	99.2	92.5	100	100	49.1	50.8	49.1	100	100	100
91.7	99.1	100	58.3	100	90.8	100	100	98.3	100	58.3	100	93.3	99.1	100	100	100	98.3	100	100	100	58.3	58.3	58.3	100	100	100
83	89	93	76	76	83	94	83	81	94	76	83	89	95	93	90	84	79	82	93	76	76	76	76	96	94	93
59.1	66.3	71.4	54.2	67	55.3	77.4	56.1	70.6	72.1	54.2	56.6	68.1	72.7	73.3	57.5	62.6	65.5	65	58.2	58.4	54.2	54.2	54.2	75.2	76.1	76.6
52	64	68	0	84	44	64	44	0	68	0	44	60	92	52	56	52	0	44	56	44	0	0	0	52	52	52
60.2	77.6	79.2	46	73.9	60.6	84.9	76	91.5	87	46	73.4	7.2	84.3	79.5	87.7	88.5	89.4	83.8	89.6	90.1	46	46	46	86.5	84.8	80.5
50	43.7	31.2	56.2	31.2	43.7	81.2	56.2	81.2	56.2	56.2	43.7	75	56.2	54.6	75	75	68.8	62.5	68.8	75	43.7	56.2	56.2	87.5	87.5	72.5
75.5	77	76.2	76.2	76.2	76.2	75.4	76.4	51.6	76.4	76.2	76.2	76.7	76.7	73.4	76.5	77.5	73.5	75.9	78.3	76.5	76.2	76.2	76.2	74.2	75.3	75
71.3	71.6	75.5	70.2	76.2	70.6	65.3	74.4	54.2	73.7	70.2	72.7	71.5	73	68.5	69.4	70.5	69.4	70.1	72.9	71.5	70.2	70.2	70.2	71.2	75.5	67.5
91.4	95.1	96.5	65.5	94.9	91.1	96.3	95.2	93.6	95.2	65.5	94.5	96.1	95.1	95.3	96.3	96	96.6	96.9	96.3	95.7	65.5	65.5	65.5	97	97	96.6
89.3	94	93.4	62.7	92.6	90.1	96.8	94.7	96.5	95.2	62.7	93.4	94.5	95	95.4	95.4	95.6	94.7	93.8	96.7	98.2	62.7	62.7	62.7	95.8	97	95.3
69.2	76.7	75.7	76.2	75.7	76.2	79	72.2	68.5	73.2	76.2	75.7	79	75.7	78.3	76.5	80	69	73	82	76	76.2	76.2	76.2	80.5	80.8	79.8
52.8	66.9	62.2	18.8	59.4	40.5	73.1	40.5	66.3	63.2	18.8	40.5	74	66.9	62.2	74	52.9	72.1	68.3	56.7	19.2	18.8	18.8	19.8	77.9	68.8	72.6
63	60.9	61.3	61.3	61.3	61.3	0	62	15.6	60.2	61.3	61.3	61.9	59.7	58.2	62.2	61.2	55.3	61.7	62.2	61	61.3	61.3	61.3	61.5	62.1	59.3
64.5	71.1	75.4	53.3	71.6	71.1	84.1	77.4	82.7	75.9	53.3	73.5	76.9	74	80.3	69.2	75	72.1	77.4	83.2	79.3	53.3	53.3	53.3	84.1	84.2	77
34.6	79.2	73.5	9	62.7	17.3	96.1	17.3	90	80.9	9	17.3	21.2	87.4	90.7	71.2	42.2	93.9	82.5	71	9.1	9	9	9	98.7	99.4	93.9
85.5	86	85.9	55.5	84.2	85.5	88.4	85	77.8	86.2	55.5	85.5	87.1	86.2	86.2	87.1	86.3	81.2	86.5	85.8	86.3	55.5	55.5	55.5	88.4	86.8	87.4
60	70.1	70.3	60.9	67.3	67.3	80.9	70.7	65.8	70.8	60.9	68.7	78.9	73.2	77.7	74.6	71.1	70.3	69.9	75.4	75.6	60.9	60.9	60.9	81.2	78.9	79.5
50.3	94.2	96.7	30.6	91.2	49.7	95.4	49.7	94.8	97.2	30.6	49.7	94.8	96.4	95.9	97.3	65.8	96.5	93.5	73.4	30.4	30.6	30.6	30.6	98.1	97.8	95.1
85.5	84.7	83.2	67.1	83.2	85.4	81.8	84.7	75.8	81.6	67.1	85.4	84.1	85.4	83.2	86.4	85.6	82.6	85.6	83.3	81.1	67.1	67.1	67.1	83.3	84	83.9
66.1	79.1	77.6	42.5	75	62.7	85.4	64.5	83	82.1	42.5	64.5	83.6	78.2	81.9	86.9	77.1	83.9	83	76.5	45.2	42.5	42.5	42.5	88.1	86.6	84.2
84.5	94	91	46.8	91	81.2	96.9	81.2	92.2	90.7	46.8	81.2	94	95	97.7	85	81.5	85.4	92.2	88.8	84.4	46.8	46.8	46.8	93.9	97.5	97.4
89.1	90.4	89.5	49.8	89.7	73.5	91.8	73.3	84.8	90.2	49.8	73.6	90.2	90.3	92.3	83.2	84.1	87	89.1	87.5	87.9	49.8	49.8	49.8	89.5	92.3	93.2
88	87	88	88	88	88	78	88	70	88	88	88	88	88	80	85	88	87	88	88	88	88	88	88	85	88	87
52.1	56.7	59.7	30.9	61.3	36.5	66.7	35	62	61.8	30.9	35	57.3	64.4	68	43.8	50.5	43.8	63.5	48.4	20.8	30.9	30.9	30.9	67.7	69.1	69.1
54.7	63.5	62.6	35.5	60.2	44.8	77.8	44.8	69	71.9	35.5	44.8	63	68.6	74.4	59.3	54.6	57.4	64.8	64.4	31.9	35.5	35.5	35.5	77.8	75.7	77
72.9	76.1	73.2	73.5	73.2	73.2	63.5	74.1	50.7	73.8	73.5	74.5	73	73.8	70.6	75	73	75	72.4	72.4	72.7	73.5	73.5	73.5	71.4	69.6	68
75	89.2	50	50	46.4	53.5	89.3	53.5	85.7	85.7	50	53.5	75	75	92.9	53.6	53.6	82.1	60.7	78.6	71.4	50	50	46.4	85.7	85.7	89.3
52.8	52.4	58	54.1	56.4	54.1	59.5	53.4	52.3	59.4	54.1	53.4	57.9	57	56.4	59.5	57.9	58.6	59.9	58.6	56.6	54.1	54.1	54.1	57.6	59.7	59.1
81.6	75.8	77.8	63.9	78.9	77.8	83.1	80.2	74.7	81.2	63.9	80.2	83.1	80.9	77.6	83.1	83.4	86.1	81.1	84.1	84.5	63.9	63.9	63.9	83.1	83	80.6
30	33.3	39	39	39	39.8	41.1	35.7	33.1	37.3	39	35.7	12.9	35.7	32.4	49.2	45.2	38.7	43.5	38.7	24.2	39	39	39	41.1	35.8	42.4
24.5	28	28	28	28	28	32.5	28	28	32.5	28	28	32.5	35	31	33.5	34	26	35	34.5	24	28	28	28	35	32.5	38.5
80	78.7	83.8	79.3	77.4	78	82.7	80.6	79.5	78.7	79.3	78	80.1	82.5	80.7	78.8	82.1	84	81.4	86.5	79.5	79.3	79.3	79.3	81.4	83.2	82.6
75	83.8	82.3	60.2	85.2	77.9	80.9	79.4	64.7	79.4	60.2	82.3	85.3	85.2	82.4	86.8	88.2	76.5	60.3	88.2	86.8	60.2	60.2	60.2	88.2	88.2	82.4
66.7	70.6	70.4	71.3	70.6	71.3	73.1	69.1	58.9	69.4	71.3	71.3	71.7	69.2	69.6	73.8	72.6	68	71.9	71.9	73.8	71.3	71.3	71.3	69.7	70.2	71.5
84.1	90.3	86.8	64.1	86.3	80.6	93.8	88.6	90.3	91.4	64.1	84.9	90.1	90.3	92.3	86.6	86.6	88.6	90.3	94.9	88.1	64.1	64.1	64.1	93.5	92.9	91.7
92	96	94	32	94	66.6	96.7	94	94.7	94.6	32	94	96.7	95.3	92.7	98.7	81.6	97.4	95.4	96.7	97.4	33.3	32	32	96.7	96	96.7
58.3	75	87.5	62.5	79.1	58.3	83.3	62.5	75	83.3	62.5	70.8	16.7	66.6	81	87.5	83.3	79.2	66.7	83.3	79.2	62.5	62.5	62.5	83.3	87.3	95.8
15.8	55.5	51.6	6.6	39.1	11.6	74.7	12.7	70.6	66.3	6.6	12.7	12.5	65.5	78.1	59.7	25.8	66.1	57.8	43.3	6.7	6.6	6.6	6.6	79.7	84.7	79.7
71.6	82.8	82.4	51.9	81.5	61.3	92.3	63.2	90.2	87	51.9	62.7	88.5	86	86.4	85.2	73.9	80.8	88.3	75.8	38.7	51.9	51.9	51.9	91.9	89.8	87.8
37.8	46.8	59.3	40.6	56.2	46.8	46.9	53.1	46.9	53.1	40.6	53.1	43.8	62.5	65.8	56.2	46.9	62.5	50	50	50	40.6	40.6	40.6	53.1	62.5	59
75.7	75.6	79.7	54.7	78.3	61.4	85.1	68.9	81.8	81	54.7	66.2	9.5	79.7	84.5	85.8	86.5	80.4	77.7	84.5	66.9	54.7	54.7	54.7	87.2	85.2	85.8
81.9	81.9	81.9	53.6	81.9	81.8	0	82.7	66.2	82.9	53.6	81.8	82	82.1	78.2	79.8	78	82	80.9	80.9	80.7	53.6	53.6	53.6	81.9	82.6	79.4
75.5	73.5	89.6	49	76.4	76.4	93.3	84.9	90.4	83.9	49	83	82.7	81.1	80.4	80.8	86.5	89.4	82.7	89.4	86.5	49	49	49	92.3	90.6	85.9
58.3	61.1	58.3	50	58.3	58.3	61.1	58.3	59.3	60.1	50	58.3	61.1	61.1	61.1	59.5	57.6	40.7	66.7	51.2	70.8	50	50	50	61.1	61.1	61.1
67.1	67.1	67.1	67.1	67.1	67.1	66.2	67.1	0	66.6	67.1	67.1	67.1	66.2	65.7	67.1	67.4	64.4	67.1	66.7	66.4	67.1	67.1	67.1	66.2	65.7	65.7
52.8	52.7	52.7	47.2	52.7	52.7	55.6	52.7	50	52.7	47.2	52.7	47.2	52.7	52.8	52.8	56.9	45.8	52.8	48.1	55.6	47.2	47.2	47.2	53.7	53.7	53.7
62.2	75	83.6	56.5	78.3	65.3	90.8	74.1	76.9	77.3	56.5	72.4	81.3	82.1	81.9	74.8	76.1	78.8	74.4	86.8	86.1	56.5	56.5	56.5	87.6	88.7	85.9
56.5	70	66.8	57.7	65.6	57.7	83	58.5	70.8	73.2	57.7	57.7	80.9	79	78.8	79.4	61.8	61	73.2	84.4	82.9	57.7	57.7	57.7	80.9	81.9	81.5
78.6	89.1	86.7	57.5	84.8	78.7	94.5	78.5	91.2	88.3	57.5	79.1	90.7	89.1	90.7	93.3	82.8	79.2	87.2	93	91.6	57.5	57.5	57.5	92.1	92	92.1
87.7	85.6	84.1	75.3	82	77.4	92.3	84.1	89.3	85.6	75.3	83.5	86.2	83.5	87.7	87.8	85.7	71.9	83.7	87.2	86.7	75.3	75.3	75.3	88.8	90.8	89.8
72.1	72.2	75.6	65.1	75	69.5	75.1	74	61.3	74.3	65.1	72.7	76.2	75	77.6	78.6	78.6	75.7	76.3	77.6	75.7	65.1	65.1	65.1	76.4	76.3	75.1
86.8	82	86.7	74.5	86.7	83.9	79.8	86.7	76	83	74.5	86.7	17.3	86.7	82.1	83.7	90.4	90.4	85.6	88.5	76.9	74.5	74.5	74.5	84.6	84.9	85
70.8	65	66	51.4	66.9	68.9	67.3	68.9	58.7	63.1	51.4	68.9	60.6	67.9	70.9	63.5	68.3	70.2	72.1	77.9	73.1	51.4	51.4	51.4	71.2	71	70.7
62	54.3	66.3	52.1	65.2	56.5	62	54.3	67.4	64.1	52.1	53.2	64.1	57.6	64.4	73.9	78.3	71.7	63	75	73.9	52.1	52.1	52.1	65.2	70.9	61.8
83.3	83.3	85.2	86.2	86.2	86.2	83.7	86.2	85.6	90.1	86.2	86.2	84.6	86.2	86.3	84.6	85.6	85.6	84.6	86.5	84.6	86.2	86.2	86.2	86.5	86.3	83.4
52.5	52.3	53.3	41.9	54.2	52.3	59.6	46.6	56.7	60	41.9	46.6	30.8	59	56.3	64.4	62.5	66.3	60.6	68.3	33.7	41.9	41.9	41.9	72.1	66.8	60
63.7	71.4	71.4	71.4	71.4	71.4	58.7	71.4	50	62.6	71.4	70.3	68.5	69.2	67.6	65.8	69.6	57.6	69.6	66.8	69.6	71.4	71.4	71.4	66.3	72.5	69.3
69	66.6	71.1	71.1	71.1	71.1	59.1	71.1	40.9	64.4	71.1	70	71.6	70	56.6	65.9	70.5	60.2	72.7	61.4	72.7	71.1	71.1	71.1	70.5	68.9	65.7
29.1	38.1	40.6	25.4	42.4	26.9	48.5	27.8	39	49.3	25.4	27.8	35.7	46	41.9	44.2	39.6	22.9	49.1	36.3	30.5	25.4	25.4	25.4	50.3	48.5	42.7
83.4	90	86.1	32.3	87.6	64.7	93.3	90.9	92.3	91.9	32.3	89	91.3	90.4	91.5	95.7	86.5	89.9	88.5	91.3	94.7	32.8	32.3	32.3	94.7	93.4	93.4
31.6	77.9	85.6	12.7	53.9	26.3	92.3	26.5	84.6	90.9	12.7	26.5	10.4	83.5	88.6	87	31.9	83	53.5	31.1	6.6	13.5	12.7	12.7	90.7	90.2	86.4
55.1	55	55	55	55	55	56.1	55	43.9	55	55	55	55.1	55	57.8	57.8	60.4	55.6	71.1	55.1	58.3	55	55	55	55.1	55.1	54.5
90.9	91.9	91.9	8	80.7	76.4	92	91.9	92	91.9	8	91.9	8	91.9	92	82.4	92	74.3	92	92	73.8	8	8	8	92	92	92
61.5	67.5	67.2	67.8	67.2	67.3	66.6	66.5	49.3	67.6	67.8	66.2	68.5	66.2	64.2	66.6	67.3	67.9	68	65.3	66.1	67.8	67.8	67.8	67.4	67.8	68.1
71.9	73.7	80.7	55.5	74	70	82.7	79.6	76.1	80.7	55.5	75.9	85.7	79.2	77.8	86	88.2	87.1	83.8	85.3	87.1	55.5	55.5	55.5	85.7	83	77.8
53.5	69.6	64.8	25.7	65.6	42	78.1	39.9	74.8	69.8	25.7	39.9	72.3	72.3	75.1	78.4	50.5	63.9	72.5	75	37.5	25.6	25.7	25.6	75.4	76.4	73.9
53	86.3	95.6	16.6	78.3	33.3	98.7	33.1	98.7	94.1	16.6	33.1	95	92	96.8	98.5	51.2	97.7	93.2	68.7	16.7	16.6	16.6	16.6	98.8	98.7	98
47	49.6	34.4	34.4	34.4	37.7	49.3	39.7	52.6	53.6	34.4	39.7	49.3	51.6	54.4	46.7	47.4	42.8	47.4	49.3	50	34.4	34.4	34.4	59.2	62.2	58.9
69.9	99.4	81.9	65.3	78.2	69.4	99.3	72.1	79.9	72.9	65.3	67.9	97	97.2	98.4	97.6	74.1	66.7	93.9	98.1	97.6	65.3	65.3	65.3	99	99.2	98.7
50	60	80	40	50	50	87.5	70	87.5	60	40	50	50	60	54.2	87.5	87.5	87.5	50	87.5	100	50	0	0	87.5	87.5	79.2
77.4	82.5	80.9	67.7	81.9	77.7	80.4	78	71.8	84.5	67.7	77	83.7	84.8	86.1	81.4	79.5	74.7	82.7	86.2	82.7	67.7	67.7	67.7	82.4	84.5	84.2
72.6	78.7	75.8	48.3	76.1	78	84.3	77	79.5	80.3	48.3	77.7	83	82.5	84.2	82.7	79.5	79.2	83.3	83.3	82.1	48.3	48.3	48.3	84.6	85.1	85.8
79.2	89.8	96.6	39.8	90.4	66.8	96	89.8	98.9	96.6	39.8	87.6	92	93.8	94.9	99.4	98.3	97.2	93.2	97.2	99.4	39.8	39.8	39.8	98.9	98.9	97.2
73.3	92	90	40.5	87.1	60.3	95	60.3	95	96	40.5	60.3	75	92	96.1	99	81	94	86	75	48	40.5	40.5	40.5	95	96.1	95.1

cforest_caret	parRF_caret	RRFglobal_caret	RotationForest_weka	RandomCommittee_weka	OrdinalClassClassifier_weka	MultiScheme_weka	MultiClassClassifier_weka	CostSensitiveClassifier_weka	Grading_weka	END_weka	Decorate_weka	Dagging_weka	LWL_weka	gaussprRadial_R	glm_R	glmnet_R	mlm_R	bayesglm_caret	glmStepAIC_caret	knn_R	knn_caret	NNge_weka	IBk_weka	IB1_weka	pls_caret	gpls_R	spls_R	simpls_R	kernelpls_R
100	100	100	100	100	100	50.8	100	50.8	50.8	100	100	97.5	100	100	100	100	100	100	100	100	100	100	100	100	100	100	74.2	100	100
100	100	100	100	100	100	58.3	100	58.3	58.3	100	100	100	100	100	100	100	100	100	100	100	100	100	100	100	100	100	84.2	99.2	99.2
76	94	94	88	96	29	76	87	76	76	39	35	84	89	84	85	92	86	76	76	86	92	96	94	94	83	0	76	79	79
54.2	77.4	73.5	73.4	69.2	54.8	54.2	51.3	54.2	54.2	74.1	71.9	60.6	57.9	62.2	25.7	68.4	52.4	48	45.8	61.9	59.1	68.1	54.8	54.8	70.1	43.1	23.7	23.2	0
52	12	52	32	80	36	0	64	0	0	48	32	52	44	44	32	48	52	60	56	48	40	68	68	68	48	44	20	12	12
86.6	85.3	81.8	90.5	78.5	81.9	46	86.2	46	46	80.9	82.4	88.4	61.7	90.5	8.3	87.3	89.4	46.1	46.1	88.3	89.9	79.2	81.1	80.4	0	7.7	34.3	84	84
56.7	66.7	81.2	62.5	62.5	50	56.2	62.5	56.2	56.2	50	0	62.5	37.5	87.5	81.2	81.2	81.2	68.8	67.9	68.8	56.7	56.2	68.7	75	0	81.2	81.2	87.5	87.5
79	74.9	73.8	77.6	70.3	77.2	76.2	77.4	76.2	76.2	77.2	77.9	76.2	76.2	78.6	76.7	77.1	76.7	77.3	76.9	77.8	79	68.3	70.3	67.2	77.3	76.7	76.2	76.9	76.9
73.4	73.1	64	72	67.1	70.6	70.2	72.7	70.2	70.2	70.6	71.6	72.7	72	75	70.1	69.8	70.1	70.3	74.5	72.6	74.8	68.8	69.9	70.2	72	70.1	69.4	69.1	69.1
95.8	96.6	96.3	97.2	95.5	95.5	65.5	96.4	65.5	65.5	95.5	95.8	96.2	91.1	95.9	96.6	96.3	96.6	96.9	96.3	96.1	96.9	96.8	95.8	95.8	96	96.4	77.7	95.9	95.9
95.6	96.8	96	97.3	96.6	92.7	62.7	94.3	62.7	62.7	92.7	96.6	95.7	91.2	97.2	94.7	96.8	94.2	97	95.4	96.7	96.7	95	94.9	94.9	96.5	92.8	74.1	96.1	96.1
79.8	80.9	81.3	78.2	75.2	73.2	76.2	76.7	76.2	76.2	73.2	74.2	76.7	76.7	79	75.5	81	75	81.3	75.7	76	77.8	76.7	65.1	65.1	82.8	75	76	79	79
67.7	71.7	67.9	69.8	73.5	65	18.8	65	18.8	18.8	68.8	70.7	57.5	44.3	72.1	34.6	70.2	63.5	28.3	27.3	75	66	70.7	69.8	69.8	70.9	23.1	34.6	38.5	38.5
61.4	62.5	60.7	59.7	58.8	61.3	61.3	60	61.3	61.3	61.3	60.2	61.3	61.6	60.8	62.2	61.9	62.2	60.7	61.4	62.8	62.3	56.7	59.5	56	59.3	62.2	61.2	62.2	62.2
80.3	82.7	78.9	81.7	82.6	75.4	53.3	70.6	53.3	53.3	75.4	83.1	76.9	72.5	78.8	67.8	73.1	70.2	75	70.6	80.3	81.7	70.6	86	86	79.3	64.9	53.8	75	75
94.2	99.4	93.9	98.4	95.4	67	9	70.5	9	9	95.6	95.2	48.7	63.4	94.2	19	82.7	81	16.9	16.9	100	98.3	94.1	100	100	57.6	17.1	22.1	18	18
85.2	87.5	86.8	86.5	86.3	84.6	55.5	86.3	55.5	55.5	84.6	85	85.6	85.5	87.5	87.6	86.3	87.6	85.7	85.5	85.9	85.2	86.5	82.3	82.3	86.1	87.5	51.2	86.2	86.2
76.2	80.3	79.3	77.9	78.5	71.2	60.9	71.4	60.9	60.9	71.2	75.1	70.8	66	73	74.4	75.6	74.4	72.3	69.3	74	75.8	69.7	68.9	68.9	74.4	74.2	41.6	75	75
97	97.8	97	98.3	97.8	91.5	30.6	95	30.6	30.6	97.5	98.6	96.4	81.9	97	71.5	97.6	96.7	47	47.3	96.7	97	95.6	95.6	95.6	97.8	45.7	57.3	67.9	67.9
85.5	84.7	84.7	83.2	82.4	84.7	67.1	84.7	67.1	67.1	84.7	87.7	83.9	85.4	85.6	80.3	82.6	80.3	80.2	80.9	82.6	84.7	83.9	75.5	75.5	85.6	80.3	85.6	86.4	86.4
83	87.2	86	85.4	83	79.7	42.5	86.3	42.5	42.5	85.7	82.4	74.1	64.5	86.6	20.5	86.6	84.2	63.1	61.9	86.9	87.2	82.1	81.5	81.5	84.8	64.3	58.6	52.1	52.1
94	97.8	97.5	97.1	96.4	96	46.8	88.5	46.8	46.8	95.7	96.4	81.5	81.2	88.8	5.5	87.4	88.5	59	63.5	91.8	89.7	95.7	77.9	77.9	84.4	63.4	65.4	87.8	87.8
90.6	92.1	92.7	91.7	92.4	90.6	49.8	88.2	49.8	49.8	90.7	89.9	83.9	84.1	85.8	4.6	84.9	87.2	73.6	74.1	87.4	90.6	92.9	79	79	85.5	73.7	73.7	82.6	82.6
88	88	85	88	86	88	88	85	88	88	88	89	88	87	88	84	84	84	86	83	85	88	86	84	84	88	84	88	88	88
62.8	70.1	69.6	63.4	65.9	55.6	30.9	43.2	30.9	30.9	66.4	65.9	47.9	45.8	52.6	20.8	42.7	30.7	30.9	33.5	44.8	46.9	56.7	47.4	47.4	55.7	36.5	28.1	21.9	21.9
68.3	78	78.5	69.6	76.1	66.8	35.5	64	35.5	35.5	71.9	72.8	51.4	45.7	67.6	37	62.5	60.2	47.3	47.2	63	68.7	68.2	70.5	70.5	60.9	46.8	52.3	48.6	48.6
74.2	67.7	69.3	73.8	64	72.8	73.5	74.8	73.5	73.5	72.8	75.1	73.8	73.2	73.4	75	74	75	74.5	73.9	74.3	76.2	68.9	65.3	64	74.2	74.7	73.4	74	74
75	92.9	89.3	46.4	89.2	89.2	50	53.5	50	50	89.2	71.4	60.7	75	64.3	64.3	53.6	53.6	60.7	60.7	85.7	85.7	82.1	85.7	82.1	39.3	60.7	50	53.6	53.6
57.4	59.4	56.4	56.7	58.4	52.4	54.1	58	54.1	54.1	55.4	57.4	58.7	57.7	60.9	19.7	59.5	59.2	58.1	56.4	59.5	58.8	52.8	53.7	53.7	60.1	58.9	61.2	49.7	49.7
83.7	82.3	81.3	79.5	78.2	76.5	63.9	81.6	63.9	63.9	76.5	79.5	81.2	76.8	85.1	83.4	82.8	83.4	83.4	81.7	83.8	84.7	77.8	79.2	79.2	82.7	83.1	66.2	85.8	85.8
41.4	41.4	39	34.9	32.5	33.3	39	34.9	39	39	33.3	34.9	31.7	34.1	43.5	43.5	53.2	50.8	38.2	37.4	41.1	48	34.9	39	39	43.9	22.6	39.5	42.7	42.7
33.5	37.4	33.5	33.5	34.5	33.5	28	30.5	28	28	37	39.5	33.5	30	32.5	23	33.5	35.5	29.5	31.5	26	38.5	30.5	33.5	33.5	35.4	34.5	40	37.5	37.5
83.2	82.6	79.3	80.6	81.2	77.4	79.3	81.2	79.3	79.3	77.4	83.2	82.5	74.8	80.8	80.8	81.4	80.8	80.7	78.7	84	85.8	77.4	83.2	83.2	81.3	81.4	30.8	80.8	80.8
75	88.2	82.4	83.8	88.2	70.5	60.2	88.2	60.2	60.2	70.5	72	91.1	77.9	86.8	88.2	91.2	88.2	86.8	91.2	83.8	83.8	82.3	73.5	73.5	85.3	86.8	61.8	86.8	86.8
71.9	68.4	69.5	69.9	68.4	71.5	71.3	73	71.3	71.3	71.5	70.1	71.3	71.3	74.5	77.4	76.4	77.4	73.4	71.7	71.1	71	68.2	65	65	71.4	77.6	71.9	71.9	71.9
90.6	93.2	92.3	94	93.4	90.5	64.1	86.3	64.1	64.1	90.5	92.8	82.6	82.9	86.6	88.4	87.5	88.4	89.5	87.5	85.5	84.6	89.1	86	86	88.3	87.2	40.3	85.8	85.8
95.3	96.7	96	96	94	94.6	32	96.6	32	32	94.6	96	87.3	94	97.4	0.7	97.4	98.7	66.7	66.7	95.4	94.7	96.6	96	96	82.7	65.1	67.8	97.4	97.4
62.6	83.3	78.9	75	83.3	62.5	62.5	83.3	62.5	62.5	75	87.5	62.5	79.1	79.2	33.3	83.3	83.3	33.2	24.6	87.5	67.6	70.8	91.6	87.5	83.3	25	62.5	66.7	66.7
71.7	82.5	78.3	84.4	75.2	48.3	6.6	57.7	6.6	6.6	73.6	77.5	50	45.2	70.3	8.3	73.3	64.7	11.9	10.3	84.7	70.6	71.9	83	83	57.8	7.8	11.9	10	10
88.1	90.4	89.5	89.4	87.9	78.3	51.9	77.7	51.9	51.9	86.2	89.4	86.6	72.8	89.1	59.8	91.5	76.3	63.5	59.3	88.3	86.8	84.3	83.6	83.6	85.7	61.3	63.7	54.1	54.1
56.6	60	50.8	62.5	53.1	53.1	40.6	50	40.6	40.6	43.7	62.5	43.7	53.1	59.4	62.5	46.9	46.9	39.7	40.1	43.8	54	68.7	50	50	62.5	56.2	43.8	59.4	59.4
79	87.9	82.4	84.4	81	75.6	54.7	79.7	54.7	54.7	75	80.4	82.4	68.2	87.8	7.4	83.1	81.8	54.7	56.1	79.1	81.7	78.3	77	77	85.8	28.4	65.5	85.1	85.1
82.9	80.9	80	82.4	76	82.6	53.6	82.5	53.6	53.6	82.6	83.6	81	81.8	81	82.3	82.1	82.3	82.6	82.3	78.8	81	73.6	74	74.8	80.5	82.3	55	79	79
87.8	88.7	81.1	84.9	82	79.2	49	79.2	49	49	79.2	87.7	83	79.2	85.6	70.2	87.5	79.8	77.2	77.4	72.1	81.3	77.3	69.8	69.8	82.1	76	73.1	82.7	82.7
71.3	61.1	61.1	57.6	61.1	72.2	50	58.7	50	50	72.2	52.7	61.5	58.3	0	58.8	60.2	58.8	59	63.9	60.6	58.8	61.1	69.4	69.4	58.8	58.8	60.2	58.8	58.8
67.1	65.7	65.7	66.6	65.7	64.3	67.1	67.3	67.1	67.1	64.3	58.7	67.1	67.1	0	67.4	67.1	67.4	67.4	67.1	64.8	64.1	65	65.2	65.2	67.1	67.4	67.1	67.4	67.4
52.8	53.7	53.7	53.7	57.4	52.7	47.2	56.2	47.2	47.2	52.7	59.7	71.7	52.7	0	56.2	56.5	56.2	56.2	52.8	55.1	55.3	61.1	54.6	54.6	53.9	56.2	72.2	53.9	53.9
84.7	89.9	87.8	88.8	88.8	81.7	56.5	84	56.5	56.5	81.7	88.8	80.2	71	85.5	73.3	80	79	86.3	75	85.3	87.2	80.2	85.5	85.5	81.1	68.9	59.7	78.6	78.6
79.8	81.4	81.7	80.7	77	73.1	57.7	80.2	57.7	57.7	73.1	78	69.9	62.1	78.7	42.1	79.9	79.8	80.3	79.1	75.2	76	68.4	74.1	74.1	77.6	9.8	66.3	75.9	24.7
88.6	92.5	91	92.7	91.5	85.7	57.5	91.2	57.5	57.5	89	90.7	86.6	78.8	90.5	73.4	92.5	91.8	56.7	56.1	90.1	89.1	84.6	90.1	90.1	92.2	0	56	67.3	26.4
86.2	89.2	90.8	91.2	90.2	84.6	75.3	87.1	75.3	75.3	84.6	90.2	85.6	83.5	84.7	84.2	85.2	86.2	86.7	85.1	92.9	90.7	86.1	93.8	93.8	87.2	84.2	37.8	87.8	87.8
77.3	76.6	76.7	75.9	73.9	73.3	65.1	76.6	65.1	65.1	73.3	74.8	73.4	72.1	78.3	78.9	78.4	78.9	77.1	77.1	78	75.7	72.6	73.3	73.3	77.2	79	65.2	78.8	78.8
86.8	84	83.1	86.7	82	85.8	74.5	81.1	74.5	74.5	87.7	86.7	85.8	86.7	85.6	18.3	82.7	80.8	17	14.2	86.5	86.8	84.9	57.5	84.9	86.9	14.4	47.1	69.2	69.2
69.8	74.8	67	67.9	68.9	66	51.4	62.1	51.4	51.4	69.9	75.7	68.9	68.9	75	18.3	68.3	67.3	33.1	37	67.3	72.8	62.1	53.3	66	66.1	33.7	37.5	40.4	40.4
67.4	65.1	70.6	67.3	67.3	64.1	52.1	66.3	52.1	52.1	64.1	66.3	63	65.2	80.4	18.5	68.5	67.4	62.1	63.1	76.1	68.5	65.2	48.9	66.3	72.7	69.6	60.9	70.7	70.7
86.3	88.2	89.2	85.2	85.2	83.3	86.2	87.2	86.2	86.2	83.3	84.3	86.2	82.3	85.6	83.7	83.7	83.7	86.3	88.3	85.6	87.3	81.3	84.3	85.2	88.3	84.6	84.6	85.6	85.6
59.3	64.9	53.3	60	54.2	49.5	41.9	56.1	41.9	41.9	63.8	62.8	53.3	56.1	69.2	30.8	57.7	55.8	18.1	20.1	66.3	57.3	55.2	32.3	56.1	61	15.4	38.5	34.6	34.6
71.4	71.4	66	71.4	63.1	71.4	71.4	68.1	71.4	71.4	71.4	70.3	71.4	71.4	69	66.8	68.5	66.8	71.4	71.4	69.6	71.4	65.3	60.4	60.4	71.4	66.3	69.6	69	69
71.1	61.2	64.6	70	61.1	68.8	71.1	61.1	71.1	71.1	68.8	71.1	71.1	68.8	70.5	43.2	62.5	59.1	65.7	68.9	72.7	71.1	53.3	56.6	54.4	70	58	70.5	25	25
48.3	49.4	43.1	44.5	38.7	35.1	25.4	40.9	25.4	25.4	49.3	43.6	45.1	34.5	50	11.6	47.9	45.1	20.6	21.2	46	45.2	36.9	35.1	34.2	50.9	21.3	15.9	9.8	9.8
91.9	94.3	94.7	93.3	91.9	85.2	32.3	93.8	32.3	32.3	89	92.8	91.4	90.9	89.9	13	93.3	93.3	63.3	62.4	90.4	93.4	89.5	92.3	92.3	96.7	63	51	74.5	74.5
79.8	89.6	0	82.1	92.5	66.7	12.7	88	12.7	12.7	84	65.1	71.5	42.8	0	21.3	91.8	85.1	5.9	5.3	83.8	82.2	88	84.8	84.8	70.2	8.2	19.9	21.3	21.3
65.8	55.1	55.6	61.4	57.7	66.8	55	59.3	55	55	66.8	63.6	55	55	0	59.4	59.4	59.4	59.9	57.8	55.6	55.1	59.3	55.6	49.7	57.2	61	55.1	57.2	57.2
92	92	92	91.9	91.9	90.9	8	88.2	8	8	90.9	91.9	91.9	91.4	0	86.1	92	73.8	92	92	91.4	92	91.9	91.9	91.9	91.4	88.2	91.4	90.4	90.4
68.1	67.7	68.7	67.1	62.6	63.6	67.8	66.6	67.8	67.8	63.6	63.3	67.6	66.2	67.7	66.4	67.6	66.4	66.4	67.1	68.3	66.2	61.1	54.9	54.9	67.7	66.6	32.1	67.4	67.4
85.2	83	79.6	80.7	77	77.4	55.5	83.3	55.5	55.5	77.4	80.3	82.9	68.5	88.2	86.4	87.1	86.4	85.6	81.5	85.7	85.9	76.2	75.5	75.5	84.8	86.4	63.6	87.1	87.1
73.4	75.8	76.5	77.4	74.2	72.3	25.7	78.8	25.7	25.7	73.6	76.7	65.4	45.3	73	51.4	79.1	80.1	49.1	48.8	70.3	70.9	60.8	69.2	69.2	74.9	49.4	37.5	48.8	48.8
97	98.3	97.3	97.3	97	84.5	16.6	92	16.6	16.6	93.5	97.8	96.8	74	98	12.7	98.8	86.8	17	16.8	97.2	97.2	97.6	96.5	96.5	83.7	17.5	39.3	38.7	38.7
58.2	62.1	57	58.9	60.9	47	34.4	52.9	34.4	34.4	49	60.2	49	47.6	50	31.6	46.7	47.4	41	42.9	55.9	56.3	62.2	55.6	55.6	56.3	34.2	35.5	34.9	34.9
97.6	99.1	99	97	96.8	93.7	65.3	98.3	65.3	65.3	93.7	95.3	81.2	70.4	87.2	97.6	97.6	97.6	98.3	98.3	100	99.5	89.7	100	100	98.4	97.6	54.3	97.6	97.6
41.7	91.7	87.5	80	70	80	40	60	40	0	80	0	40	70	87.5	50	87.5	87.5	87.5	50	75	66.7	70	70	70	0	87.5	75	87.5	87.5
83.9	85.2	83.5	87.4	83.8	80.3	67.7	84.5	67.7	67.7	80.3	80.9	70.3	77.7	84.6	85.9	86.5	86.9	84.2	86.1	79.5	82.2	80.9	79	79	84.2	85.9	66.7	82.1	82.1
83.6	86.4	83.9	83.8	84.1	81.2	48.3	84.8	48.3	48.3	82.5	81.9	71.2	77.7	82.4	63.5	86.5	85.6	39	39	75	80.3	76.4	77.7	77.7	81.9	39.4	43.6	67.3	67.3
97.8	98.9	94.9	97.7	95.5	93.2	39.8	97.1	39.8	39.8	93.2	97.7	96.6	89.3	98.3	1.7	99.4	98.9	73	70.8	97.2	96.6	93.8	94.9	94.9	98.9	70.5	56.2	92.6	92.6
88.2	98.1	97.1	94	97	94	40.5	95	40.5	40.5	93	94	78.2	89.1	93	1	95	96	60.4	60.4	96	93.2	95	96	97	93.9	64	83	87	87

widekernelpls_R	SimpleLogistic_weka	Logistic_weka	multinom_caret	mars_R	gcvEarth_caret	pam_caret	VFI_weka	HyperPipes_weka	Vote_weka	FilteredClassifier_weka	CVParameterSelection_weka	ClassificationViaClustering_weka	AttributeSelectedClassifier_weka	ClassificationViaRegression_weka	KStar_weka	svmlight_C	dpp_C
100	100	100	100	100	100	100	98.3	50.8	50.8	100	50.8	71.6	100	100	100	100	100
99.2	100	100	100	100	100	92.4	91.6	83.3	58.3	100	58.3	64.1	100	100	100	100	100
79	92	79	92	85	88	83	55	76	76	89	76	2	80	92	95	91	12
23.2	72.3	57.3	50.2	30.1	68.8	71.5	47.3	62.8	54.2	70.7	54.2	28	69.9	65.9	51.1	67.7	52.2
12	76	64	52	12	12	52	64	0	0	68	0	52	72	76	76	72	76
84	88	89.4	88.8	83.2	91.2	88.2	66	7.8	46	75	46	56.3	78.8	88.6	89.2	91	87.7
87.5	31.2	62.5	64.2	25	44.2	56.7	75	56.2	56.2	56.2	56.2	18.7	43.7	50	68.7	93.8	62.5
76.9	77.1	77.4	77.1	77.1	78.1	76.6	37.4	76.2	76.2	76.2	76.2	58.5	75.6	77.8	74.3	77.5	65.1
69.1	73.7	72.7	69.9	70.1	76.2	70.6	68.1	70.2	70.2	73	70.2	72.3	72.7	72.7	71.3	70.4	71.5
95.9	96.1	96.4	96.3	96.7	96.3	95.4	92.1	88.9	65.5	94.8	65.5	95.7	95.5	95.7	95.1	97.1	96.7
95.4	97.1	94.3	97.5	95.1	94.7	93.7	91.9	93.1	62.7	94.3	62.7	92.6	93.4	94.3	94.5	96.3	95.2
79	74.7	76.7	79.8	74.5	70.3	77.3	61.6	75.2	76.2	76.2	76.2	59.5	76.2	74.7	63.6	77	55.1
45.2	66.9	67.9	70.1	50	62.1	58.4	62.2	61.3	18.8	56.6	18.8	44.3	66.9	66.9	68.8	68.3	61.5
62.2	60.4	60	58.4	62.4	62.8	61.4	52.4	61.3	61.3	61.3	61.3	57.7	61.6	61.1	60.9	58.9	56.2
75	74.5	70.6	75	72.6	66.3	72.6	59.1	63.9	53.3	73.5	53.3	55.2	78.3	79.8	82.2	88	64.9
18	82	80.9	81	30.5	76	62.1	71.4	49.1	9	74.4	9	31.6	76.6	82.9	100	100	49.8
86.2	84.9	86.3	85.8	86.6	86.9	84.2	78.6	44.4	55.5	85.6	55.5	70.5	85.6	86.2	77.9	86.6	85.9
75	70.7	71.4	72.7	76.2	71.9	66.2	67.7	46	60.9	67.5	60.9	56.8	67.3	71	76.7	73.4	68.6
66	97.2	97.5	96.7	71.2	95.4	96.2	93.1	90.1	30.6	96.1	30.6	74.5	95.9	97.2	94.5	95.6	97
86.4	85.4	84.7	80.1	82.6	80.9	82.4	83.2	83.9	67.1	85.4	67.1	70.9	83.2	85.4	71.7	85.6	75.8
52.1	86.9	83.9	87.2	64	82.4	80.4	74.7	70.2	42.5	77.3	42.5	56.5	78.2	85.7	80.6	88.4	84.2
87.8	88.6	88.1	87.9	86.8	95.6	81.2	81.2	47.3	46.8	90.1	46.8	63.6	93.4	95	95.5	93	84.4
82.6	86.7	89.4	89.5	86.6	91.3	88.5	81.3	50.3	49.8	88.2	49.8	51	89.4	90.3	92.5	90.4	84.2
88	88	85	86	88	86	88	59	86	88	88	88	49	88	87	83	86	46
21.9	52	37.6	46.5	20.8	66	50	44.3	32.9	30.9	61.3	30.9	27.3	67.5	59.2	55.1	47.4	47.4
48.6	62.1	60.2	65	49.5	67.4	55.6	57	50.4	35.5	64	35.5	36.9	70.5	71.4	72.8	67.9	58
74	74.1	74.8	74.2	73.4	74.5	73.5	59.8	73.2	73.5	73.2	73.5	48.6	73.2	73.2	70.2	71.7	70.7
28.6	60.7	53.5	53.6	42.9	85.7	64.3	75	53.5	50	53.5	50	21.4	89.2	57.1	85.7	82.1	3.6
49.7	59	58	58.1	53.9	56.4	57.1	45.2	54.4	54.1	55.1	54.1	30	59.7	58	52.8	57.2	54.6
85.8	80.6	81.6	82.3	83.1	82.3	82.3	83.3	64.9	63.9	77.5	63.9	81.6	76.1	78.2	75.5	80.8	85.3
31.5	34.1	39	40.7	37.9	39.1	43.1	26.8	30.8	39	39	39	26	35.7	42.2	33.3	21	34.7
37.5	34.5	31.5	32.1	31.5	30.5	33.5	32	23.5	28	28	28	24	33.5	31	31	28	33
80.8	80	81.2	83.3	80.1	80	80.7	72.2	60.6	79.3	80	79.3	67.7	76.7	80	76.7	82.7	72.4
86.8	91.1	88.2	88.2	82.4	80.9	83.8	79.4	60.2	60.2	86.7	60.2	58.8	86.7	83.8	75	83.8	60.3
71.9	71.5	73	72.7	73.6	71.2	71.4	47.6	72.5	71.3	69.6	71.3	58.4	71.5	71	61.9	71.2	55.8
85.8	87.4	86.3	85.5	88.6	83.2	84	93.4	35.8	64.1	87.1	64.1	69.5	90.5	90	81.1	93.8	83
97.4	95.3	95.3	97.3	98.7	95.2	93.3	94.6	92.6	32	94	32	88	95.3	95.3	94.6	95.9	83.1
66.7	79.1	79.1	83.1	75	74.5	62.6	70.8	79.1	62.5	58.3	62.5	41.6	79.1	79.1	70.8	83.3	75
10	76.1	66.9	64.7	15.8	67.2	57.8	56.6	56.9	6.6	54.7	6.6	33.6	64.4	63.3	80.5	80	47.8
54.1	90.5	79.4	91.7	56.6	81.6	76.8	78.5	73.4	51.9	84.3	51.9	38	83.4	87.1	87.1	89.8	74.2
59.4	62.5	40.6	53.9	40.6	47.6	59.8	50	75	40.6	50	40.6	40.6	53.1	62.5	46.8	46.9	40.6
85.1	79.7	79.7	85.8	79.1	80.4	82.5	81	54	54.7	70.2	54.7	39.1	72.9	80.4	81.7	84.5	77
79	82.3	82.5	82.6	80.8	83	80	60.7	55	53.6	82.1	53.6	78.9	82.2	83.5	80.9	80.8	80.6
82.7	81.1	79.2	72.7	87.5	87.7	83	66.9	69.8	49	86.7	49	65	83.9	79.2	80.1	79.8	78.8
58.8	63.8	58.7	60	58.3	58.3	60.2	60.6	41.6	50	58.3	50	50	66.6	61.1	61.1	63.9	64.8
67.4	67.1	67.3	67.1	67.1	67.1	67.1	66.6	67.1	67.1	67.1	67.1	37.7	67.1	67.1	65.7	65.5	46.8
53.9	56.9	56.2	59.3	52.8	52.8	52.8	52.7	47.2	47.2	52.7	47.2	52.7	52.7	52.7	57.4	69.9	77.3
78.6	82.1	84	83.8	80.3	81.9	73.7	80.2	72.6	56.5	84.6	56.5	54.2	79.4	83.1	72.2	88.9	73.7
75.9	76.5	80.2	80.8	84	80.8	60.5	57	56.7	57.7	68.5	57.7	48.2	70.6	80	72.9	77.9	57.8
67.3	92.6	91.3	93	78.8	93.4	78.7	72.1	78	57.5	86.8	57.5	77.6	88.1	92.1	88.9	91.4	75.4
87.8	84.1	87.1	88.7	90.3	88.7	82.6	72.8	73.8	75.3	85.6	75.3	54.3	88.2	83.5	91.7	94.4	69.4
78.8	76.6	76.6	77.6	78.5	77.7	71	58.2	65.7	65.1	75.2	65.1	62.7	73.1	77.2	70.4	78.4	71.1
69.2	86.7	79.2	84.9	71.2	86.8	79.3	81.1	64.1	74.5	86.7	74.5	63.2	85.8	86.7	83.9	0	79.8
40.4	63.1	60.1	62.2	35.6	70.9	68.1	49.5	37.8	51.4	68.9	51.4	47.5	68.9	66.9	69.9	0	56.7
70.7	69.5	63	70.5	60.9	65.3	63	66.3	36.9	52.1	59.7	52.1	56.5	63	67.3	67.3	0	59.8
85.6	85.2	87.2	88.3	80.8	88.2	86.3	62.7	86.2	86.2	86.2	86.2	65.6	86.2	85.2	86.2	0	55
34.6	55.2	50.4	58	33.7	53.5	49.5	38	31.4	41.9	50.4	41.9	26.6	58	59	56.1	0	52.9
69	71.4	68.1	69.8	69.6	71.4	71.4	41.2	68.1	71.4	71.4	71.4	54.3	71.4	71.4	60.4	69.4	46.7
25	71.1	60	66.7	0	71.1	71.1	28.8	71.1	71.1	71.1	71.1	35.5	71.1	67.7	63.3	70.5	31.8
9.8	46.9	38.1	44.6	10.7	44.2	44	33	25.4	25.4	42.1	25.4	51.1	42.4	46.9	40.3	48.8	50.9
74.5	95.2	93.3	96.7	82.7	91.9	90	86.6	81.4	32.3	90.9	32.3	89	89	94.7	90.9	91.3	85.1
21.3	90.9	86.1	90.2	15.2	90.4	83.8	71	11.1	12.7	89.8	12.7	30.8	80.3	91.7	87.5	89.6	53.2
57.2	58.8	59.3	62	57.8	57.8	57.2	55	57.7	55	55	55	48.3	57.7	58.2	55.6	54.5	48.4
90.4	91.4	88.2	62.6	75.9	85.6	92	91.9	91.4	8	91.9	8	91.9	91.9	90.9	91.4	92	53.5
67.4	67.8	66.6	66.7	67.3	68	67.8	49.5	33.3	67.8	67.8	67.8	65.7	66.9	67.2	58.6	67	53.2
87.1	83.3	83.3	83.3	84.2	82.6	84.8	77	55.5	55.5	80.7	55.5	75.5	77.4	78.1	76.2	87.7	86.6
48.8	78	80.2	79.7	62	78.4	44.9	53.1	38	25.7	67.6	25.7	33.8	67.1	79.1	70.9	71.6	51.5
38.7	98.5	81.5	98.7	52.3	94.2	90	93.5	96.1	16.6	90.1	16.6	61.3	90.1	93	96.3	98.7	85.8
34.9	52.3	52.9	50.3	35.5	43.2	56.3	45.6	39.7	34.4	34.4	34.4	43	39	49.6	56.9	48	55.3
97.6	97.5	98.3	98.3	97.6	98	65.3	70.9	34.6	65.3	69.9	65.3	59.8	80.1	94.4	99	100	63.3
87.5	50	60	83.3	75	75	83.3	70	80	40	50	0	40	50	60	60	0	87.5
82.1	85.1	84.5	85.8	84	83.3	72.3	74.8	67.4	67.7	81.9	67.7	66.7	81.2	85.8	81.2	84.7	69.2
67.3	85.1	84.1	86.5	79.5	84.5	70.3	73.5	66.7	48.3	75.8	48.3	44.1	76.7	84.8	80.3	0	76.9
92.6	98.3	96.6	97.8	93.2	99.4	97.2	96.6	89.3	39.8	88.2	39.8	95.5	91.5	93.2	98.3	97.7	93.8
87	96	96	96	85	95	95.1	91	94	40.5	95	40.5	59.4	90	95	96	95	93
